# Five-year changes in health-related quality of life after proton therapy in a primarily meningioma cohort: a prospective study

**DOI:** 10.1007/s11060-026-05767-1

**Published:** 2026-08-21

**Authors:** Per Fessé, Linda Åkeflo, Karin Ahlberg, Emelie Blomqvist, Per Fransson, Ingrid Kristensen, Kristin Kunni, Ulrica Langegård, Emma Ohlsson-Nevo, Katarina Sjövall

**Affiliations:** 1 Institute of Health and Care Sciences, Sahlgrenska Academy, University of Gothenburg, Box 457, Gothenburg, SE- 405 30 Sweden; 2 Department of Oncology, Sahlgrenska University Hospital, Gothenburg, Sweden; 3 Department of Oncology, Faculty of Medicine and Health, Örebro University, Örebro, Sweden; 4 Centre for Research and Development, Uppsala University/Region Gävleborg, Gävle, Sweden; 5 Department of Nursing, Umeå University, Umeå, Sweden; 6 Radiation Physics, Department of Haematology, Oncology and Radiation Physics, Lund University Hospital, Lund, Sweden; 7 Skandion Clinic, Uppsala, Sweden; 8 Faculty of Health Sciences, Kristianstad University, Kristianstad, Sweden

**Keywords:** Proton beam therapy, Meningioma, Patient-reported outcomes and Health-related quality of life

## Abstract

**Purpose:**

Long-term patient-reported outcomes after Proton beam therapy (PBT) remain insufficiently described for patients with benign intracranial and seller-region tumors. The aim of this study was to evaluate health-related quality of life (HRQoL) and brain tumor–specific symptoms over five years after PBT in a primarily meningioma cohort.

**Methods:**

In this prospective cohort study, adults treated with PBT completed the European Organisation for Research and Treatment of Cancer (EORTC) QLQ-C30 and QLQ-BN20 questionnaires from baseline through 5 years. Mixed models for repeated measures estimated mean scores and change from baseline with 95% confidence intervals. Baseline-to-five-year change analyses included participants with available data at both time points. Exploratory analyses evaluated associations between sociodemographic factors and changes over five-years.

**Results:**

Seventy-seven of 126 participants (61%) completed the 5-year assessment. Global health status did not change significantly, whereas the QLQ-C30 summary score declined modestly. Clinically meaningful worsening was observed for fatigue and insomnia, with smaller increases in pain, dyspnoea, and constipation. Physical and cognitive functioning declined, while emotional functioning improved. On the QLQ-BN20, hair loss increased to a clinically meaningful extent and drowsiness, bladder control difficulties, motor dysfunction, communication deficits, and leg weakness worsened, whereas headaches decreased. No associations were observed between the investigated sociodemographic factors and change in fatigue or insomnia.

**Conclusions:**

Five-year outcomes demonstrated a mixed pattern with stable global health status despite a modest decline in overall HROoL and worsening in several symptom and functioning domains. Clinically meaningful increases in fatigue and insomnia, alongside smaller functional and brain tumor-specific changes, support structured long-term follow-up and rehabilitation. These findings reflect long-term outcomes following PBT, while treatment-specific contribution remains uncertain.

**Supplementary Information:**

The online version contains supplementary material available at https://doi.org/10.1007/s11060-026-05767-1.

## Introduction

Benign intracranial and sellar-region on tumors include meningiomas tumors include meningiomas, vestibular schwannomas, pituitary adenomas and craniopharyngiomas. Meningiomas are among the most common primary intracranial tumors in adults and occur more frequently in women. In Sweden, 336 new cases of meningioma were reported in 2023 [1]. Although survival is generally favourable, the term benign does not imply absence of morbidity. Tumor location and the effects of surgery and radiotherapy may contribute to persistent neurological, cognitive and functional symptoms that affect health-related quality of life (HRQoL) [2, 3]. Long-term meningioma survivors report impairments across physical, emotional, cognitive and social domains [2, 3]. Studies based on clinicians’ assessments of late side-effects report long-term toxicity among 54% of patients treated with proton beam therapy (PBT) for meningioma [4].

Treatment decisions are individualized. Observation with clinical and radiological follow-up is appropriate for many incidental, asymptomatic or radiologically stable meningiomas, while surgery is generally considered for growing or symptomatic tumors when resection is feasible. Fractionated radiotherapy or radiosurgery can be used for residual, recurrent or progressive disease, higher-risk histology, surgically inaccessible lesions, or when surgery is not appropriate [5, 6]. Compared with photon radiotherapy, PBT can reduce integral dose and exposure of uninvolved brain and adjacent critical structures, which is relevant for patients with long anticipated survival [5, 7–10]. While conventional photon radiotherapy remains an essential component of meningioma treatment, unavoidable irradiation of surrounding tissues carries the risk of late effects that can compromise function and HRQoL. PBT, with its favourable dose-distribution, is therefore of particular interest in benign tumors with long anticipated survival and lesions adjacent to critical structures [3–5, 9–11]. However, despite evidence indicating tumor control comparable to advanced photon techniques and the potential for reduced late toxicity, restricted availability, range uncertainty and limited comparative clinical evidence remain important limitations [9–11]. Short-term studies, often including mixed malignant and benign cohorts, suggest that overall HRQoL can remain relatively stable during and shortly after PBT, while specific symptoms (e.g., fatigue and insomnia) can increase in the acute and early post-treatment phases [8, 12–14]. Published photon-based meningioma series and long-term survivorship reviews show that HRQoL impairment can persist after treatment, while heterogenicity and the scarcity of comparative cohorts make it difficult to separate the effects of tumor, surgery, radiotherapy, ageing and comorbidity [3, 15]. Long-term patient-reported outcomes after PBT remain less well characterized, including for benign intracranial tumors [4, 11–13]. Therefore, the aim of this study was to evaluate five-year follow-up of HRQoL and brain tumor-specific symptoms in a cohort consisting of primarily individuals with previous meningioma treated with PBT.

## Materials and methods

### Study design and reporting

This prospective, longitudinal observational cohort study was conducted within the ProtonCare research group’s program [16] and is reported in accordance with the STROBE guidelines [17, 18]. ProtonCare is a multicentre initiative evaluating patient-reported short- and long-term symptoms and health-related quality of life following PBT.

### Setting and participants

Participants were treated with PBT at the Skandion clinic (Uppsala, Sweden), which is collaboratively managed by the seven Swedish regions university hospitals’ radiotherapy departments. Adults (≥ 18 years) enrolled in the benign brain tumor cohort treated at the clinic between August 2015 and October 2018 who were able to complete the Swedish-language questionnaires were eligible. Clinical diagnoses were retrieved from medical records and classified using ICD-10 codes.

All participants received written and oral information about the study and provided informed consent before data collection. The study was approved by the Research Ethics Committee in Gothenburg, Sweden (Dnr: 433 − 15).

### Data collection and follow-up

Patients were identified through local site contacts and invited to participate in the study by the study team. Clinical diagnosis information was retrieved from medical records, and demographics were self-reported via questionnaires. Patient-reported outcomes were collected at baseline (pre-treatment), during treatment (mid-treatment and end of treatment), and post-treatment follow-ups at 1, 3, 6, 9, 12, 24, 36, and 60 months (5 years). Questionnaires were delivered electronically or by post, and non-responders received reminder notices.

### Patient-reported outcome measures

HRQoL was assessed using the European Organisation for Research and Treatment of Cancer (EORTC) QLQ-C30 [19] and the brain tumor–specific module QLQ-BN20 [20]. Scores were linearly transformed to 0–100 scale according to the EORTC manual [21]. Higher scores indicate better functioning on the functional and global health scales and greater symptom burden on the symptom scales [19–21]. The QLQ-BN20 supplements the QLQ-C30 by assessing brain tumor–specific symptom domains by assessing four multi-item scales and seven single items. We computed the QLQ-C30 summary score [21] and interpreted change magnitudes using established thresholds and minimum important difference (MIDs), including brain-tumor–specific MIDs for group-level change [22, 23].

### Statistical analysis

Descriptive statistics are presented as mean (SD), median, minimum and maximum, and number of observations for continuous variables and as counts and percentages for categorical variables.

Pairwise change analyses included participants with available data at baseline and the 5-year follow-up. Mean change with 95% confidence intervals (CI) was estimated using inversion of Fisher’s non-parametric permutation test. Paired t-tests were used for hypothesis testing, and effect sizes were calculated using Cohen’s method (as the mean difference divided by the baseline SD).

Changes in EORTC QLQ-C30 and QLQ-BN20 scores over time were additionally analysed using mixed models for repeated measures (MMRM), using available repeated observations to estimate mean scores and differences from baseline across follow-up. Completion of every assessment, including the five-year follow-up, was not required for a participant to contribute to the MMRM. The score at each visit was modelled as a function of visit, with the corresponding baseline score included as a covariate. Within-subject correlation was accounted for using a first-order autoregressive [AR(1)] covariance structure. Adjusted mean scores and pairwise comparisons between visits were estimated from least-squares means. Degrees of freedom were calculated using the Kenward-Roger method. All available observations were included in the analysis, and missing data were handled using likelihood-based estimation under the assumption that data were Missing At Random (MAR). Contrasts for baseline-to-60-month change by sex, age category, marital status, occupation status and education category were examined within the MMRM framework.

To explore factors associated with five-year outcomes and baseline-to-five-year change, univariable linear regression models were fitted with age, sex, civil status, occupation status, and education as candidate predictors. Results are reported as beta coefficients with 95% CI, p-values, and R².

For interpretation, between-group or within-patient differences of approximately 5–10 points on EORTC 0–100 scales were considered potentially clinically relevant (small-to-moderate), whereas changes of at least 10 points were considered clearly meaningful, depending on domain and context [23].

All tests were two-sided, and *p* < 0.05 was considered statistically significant. Analyses were conducted using SAS version 9.4.

## Results

### Study participant and follow-up

**Fig. 1:**
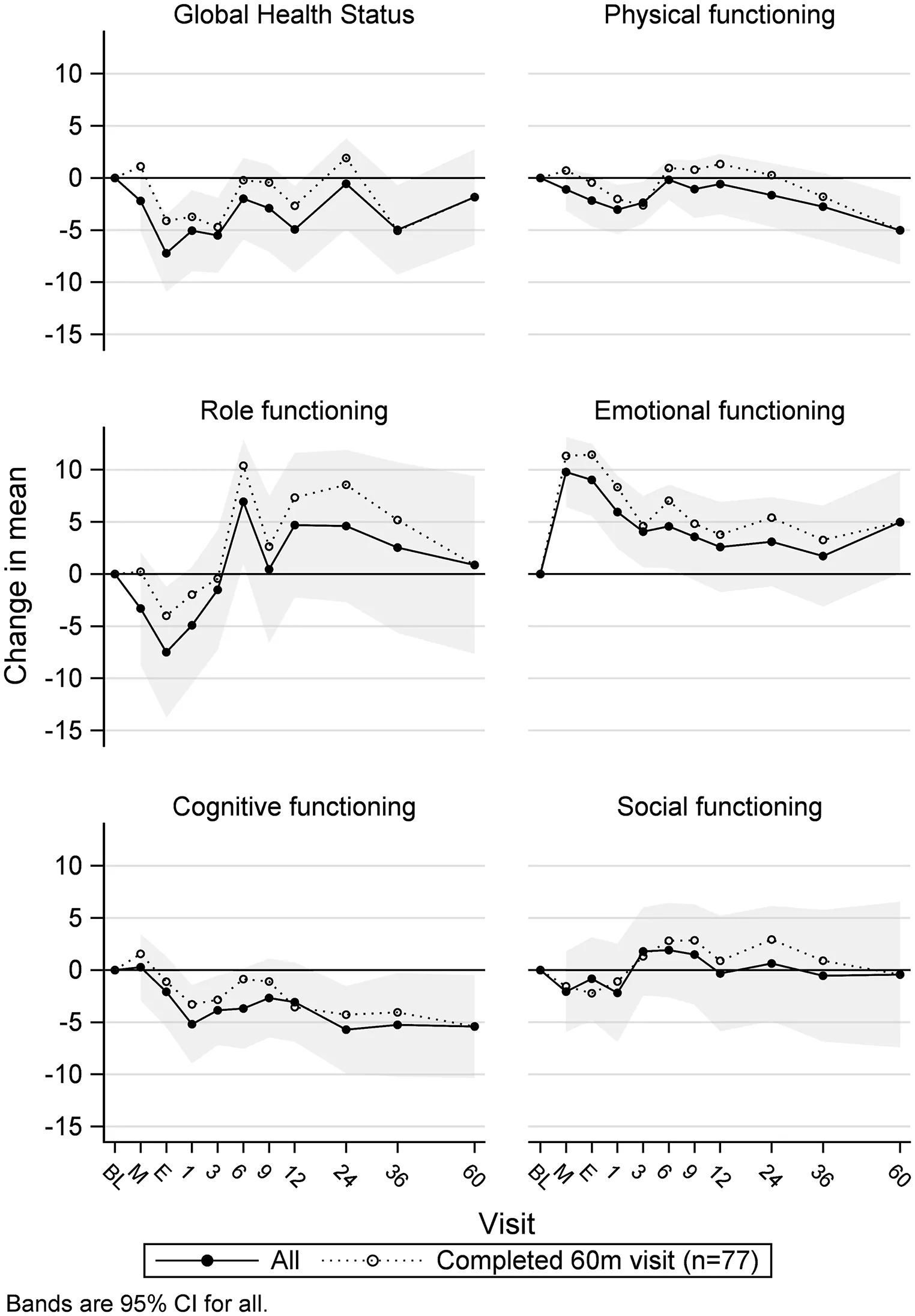
Change in EORTC QLQ-C30 global health status and functional scales from baseline through 60 months. Higher global and functioning scores indicate better outcomes, therefore, positive change indicates improvement and negative change indicates deterioration. Points show estimated mean change and bans show 95% confidence intervals

**Fig. 2:**
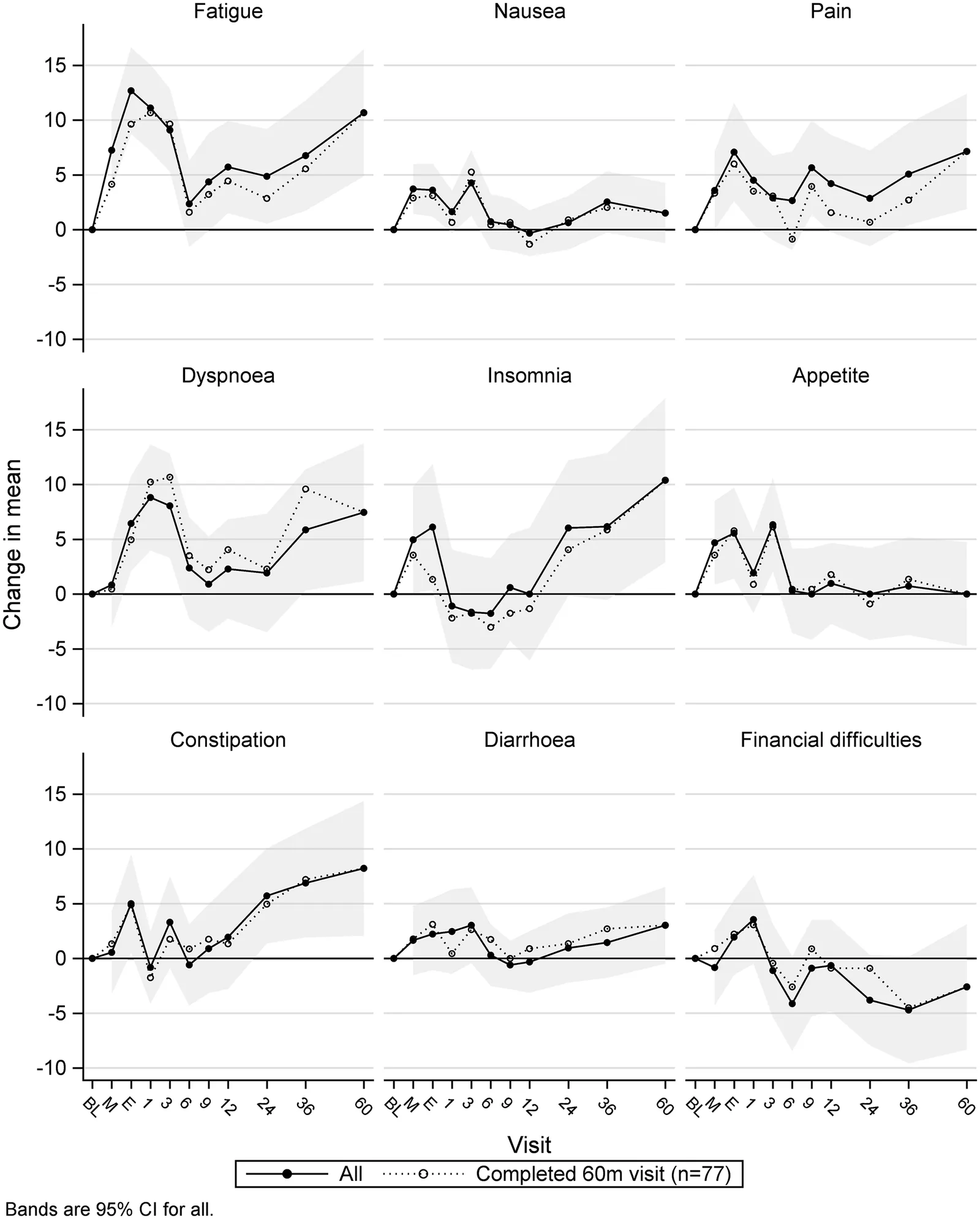
Change in EORTC QLQ-C30 symptom scales from baseline through 60 months. Higher symptom scores indicate greater symptom burden, therefore positive change indicates worsening and negative change indicates improvement. Points show estimated mean change and bans show 95% confidence intervals

**Fig. 3:**
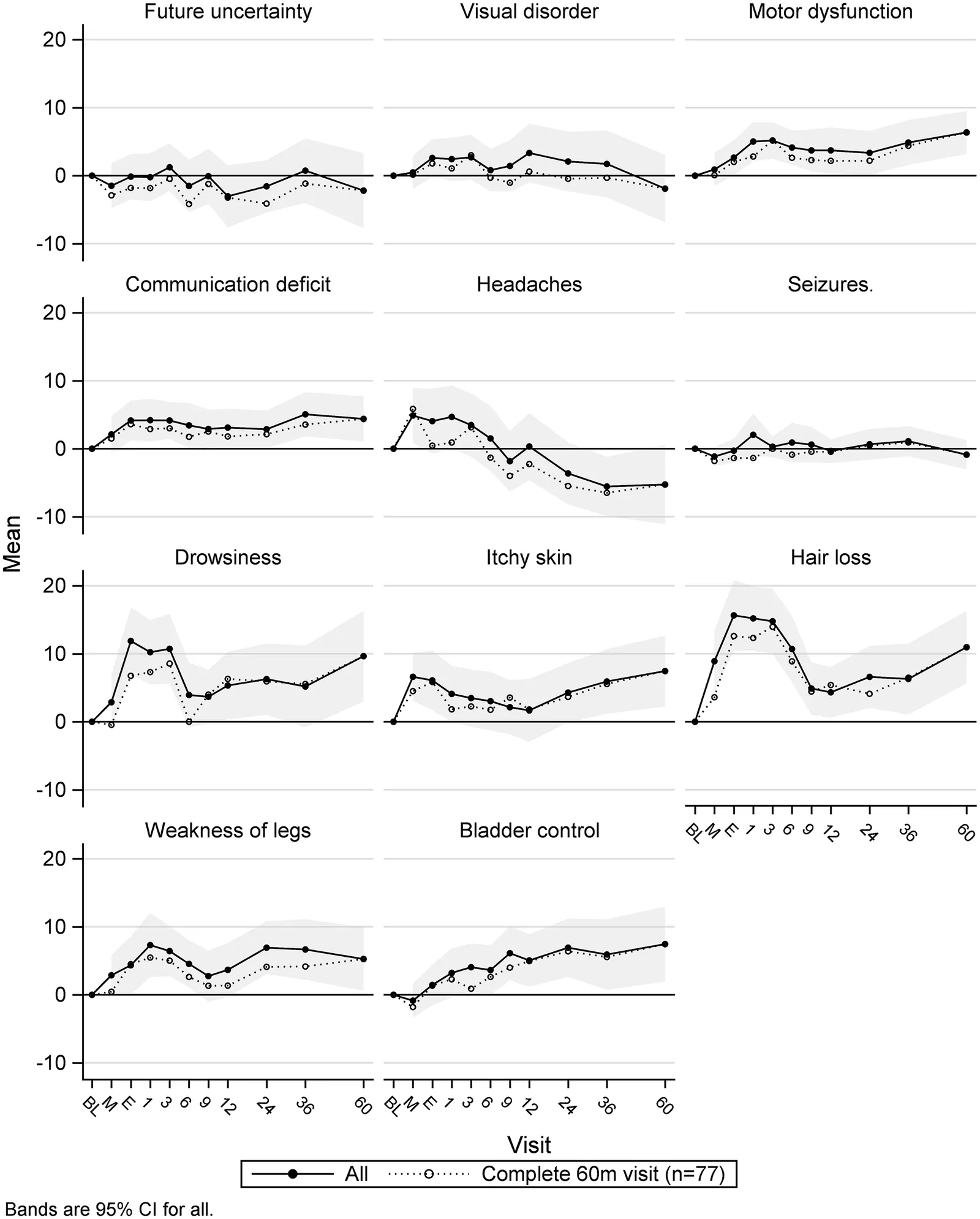
Change in EORTC QLQ-BN20 symptom scales from baseline through 60 months. Higher symptom scores indicate greater symptom burden, therefore positive change indicates worsening and negative change indicates improvement. Points show estimated mean change and bans show 95% confidence intervals

Demographic data and clinical characteristics of the study participants are presented in Table 1. In total, 126 adults completed baseline questionnaires of whom 77 (61%) completed 5 year assessment and 49 did not. Among the non-responders, 6 were not alive at the time of follow-up (the causes of death were unknown). Nonresponders at five years could nevertheless contribute earlier self-reported data to the MMRM (Figs. 1, 2 and 3). Observed baseline-to-5-year changes and change-score regression analyses were restricted to participants with data at both time points. At baseline, participants without five-year follow-up were slightly younger than responders (53.4 vs. 56.7 years) and included higher proportions of men (38.8% vs. 31.2), individuals living alone (36.7% vs. 23.4%), those with compulsory school as highest education level (16.3% vs. 11.8%), and those diagnosed with benign neoplasms other than neoplasms of the meninges (18% vs. 1.3%). Overall survival remained high over five years (Supplementary Fig. 1).

**Table 1:** Baseline demographic and clinical characteristics of the total cohort and according to 5-year follow-up status

Baseline characteristic	Total cohort (*n*=126)	Completed 5-years follow-up (*n*=77)	Lost to follow-up by 5 years (*n*=49)
**Age** (mean, (SD)) (median (range))	55.3 (13.6) 56 (22-80)	56.5 (12.7) 57 (28-74)	53.4 (14.8) 53 (22-80)
**Sex**			
Male	43 (34.1%)	24 (31.2%)	19 (38.8%)
Female	83 (65.9%)	53 (68.8%)	30 (61.2%)
**Marital status**			
Married / Cohabiting (living with a partner)	90 (71.4%)	59 (76.6%)	31 (63.3%)
Living alone (single household)	36 (28.6%)	18 (23.4%)	18 (36.7%)
**Employment status**			
Employed / Student	85 (67.5%)	50 (64.9%)	35 (71.4%)
Retired / Disability pension / Unemployed / No occupation	41 (32.5%)	27 (35.1%)	14 (28.6%)
**Education level**			
Compulsory school	17 (13.6%)	9 (11.8%)	8 (16.3%)
Upper secondary / Vocational school	60 (48.0%)	36 (47.4%)	24 (49.0%)
University / Higher education (College)	48 (38.4%)	31 (40.8%)	17 (34.7%)
**Diagnosis (ICD-10)**			
Benign neoplasm of meninges (D32.x)	107 (84.9%)	69 (89.6%)	38 (77.5%)
Benign neoplasm of brain/other CNS (D33.x)	10 (7.9%)	1 (1.3%)	9 (18.3%)
Benign neoplasm of thyroid gland (D34.0)	3 (2.4%)	2 (2.6%)	1 (2.0%)
Benign neoplasm of pituitary gland (D35.2)	3 (2.4%)	3 (3.9%)	0 (0.0%)
Benign neoplasm of craniopharyngeal duct (D35.3)	2 (1.6%)	2 (2.6%)	0 (0.0%)
Neoplasm of uncertain behaviour of pituitary gland (D44.3)	1 (0.8%)	0 (0.0%)	1 (2.0%)

All characteristics refer to baseline. Participants completing the 5-year assessment are presented separately from those lost to follow-up by 5 years.

For categorical variables n (%) is presented.

For continuous variables Mean (SD) / Median (Min; Max) / n= is presented.

### Health-related quality of life: EORTC QLQ-C30

Global health status decreased slightly compared with baseline, although the change was not statistically significant (− 2.2, 95% CI − 5.8 to 1.3; *p* = 0.22) (Fig. 1, Supplementary Table 2.1.4). In contrast, the QLQ-C30 summary score declined by − 4.4 points (95% CI − 6.1 to − 2.7; *p*<0.001), which was statistically significant but below the prespecified 10-point threshold for clearly clinical relevance.

Compared with baseline, participants reported worsening in several symptoms at five years (Figs. 2; Supplementary Table 2.1.4). In the MMRM analyses, fatigue increased by + 11.1 points (95% CI 7.4 to 14.8; *p*<0.001) and insomnia by + 10.6 (95% CI 5.4 to 15.8; *p*<0.001), representing a clearly clinically relevant worsening. Smaller increases were observed for pain (+ 8.1, 95% CI 4.4 to 11.8; *p*<0.001, constipation (+ 8.0, 95% CI 4.0 to 12.0; *p*<0.001) and dyspnoea (+ 5.9, 95% CI 1.6 to 10.3; *p* = 0.008) with changes of approximately 5–10 points. Regarding functioning domains, physical functioning declined by − 5.7 points (95% CI − 7.9 to − 3.5; *p*<0.001) and cognitive functioning by − 6.0 points (95% CI − 9.5 to − 2.6; *p*<0.001), with both changes falling within the range considered both statistically significant and potentially clinically relevant. Emotional functioning improved modestly by + 4.3 points (95% CI 1.1 to 7.4; *p* = 0.008).

Financial difficulties remained low, with no statistically significant change (− 3.4 points, 95% CI − 7.3 to 0.4; *p* = 0.079). In exploratory MMRM analyses, a greater reduction in reported financial difficulties was observed in the lower-education group (between-group difference 9.6 points, 95% CI 0.4 to 18.8; *p* = 0.041).

### Brain-tumor–specific symptoms: EORTC QLQ-BN20

At five years, several QLQ-BN20 domains worsened compared with baseline (Fig. 3; Supplementary Table 2.2.4). Hair loss increased by 11.4 points (95% CI 7.1 to 15.7; *p*<0.001) reaching the threshold for clearly clinically relevant change. Smaller increases were observed for drowsiness (+ 9.3, 95% CI 4.6 to 14.1; *p*<0.001), bladder control difficulties (+ 7.5, 95% CI 3.7 to 11.4; *p*<0.001), motor dysfunction (+ 6.7, 95% CI 4.1 to 9.3; *p*<0.001), communication deficits (+ 5.0, 95% CI 2.5 to 7.5; *p*<0.001), itchy skin (+ 7.6, 95% CI 3.7 to 11.5; *p*<0.001) and weakness of legs (+ 6.3, 95% CI 2.3 to 10.2; *p* = 0.002). Headaches decreased by 5.0 points (95% CI − 9.5 to − 0.5; *p* = 0.028). Future uncertainty, seizures and visual disorder showed no evidence of long-term group-level change.

### Associations with sociodemographic factors

**Fig. 4:**
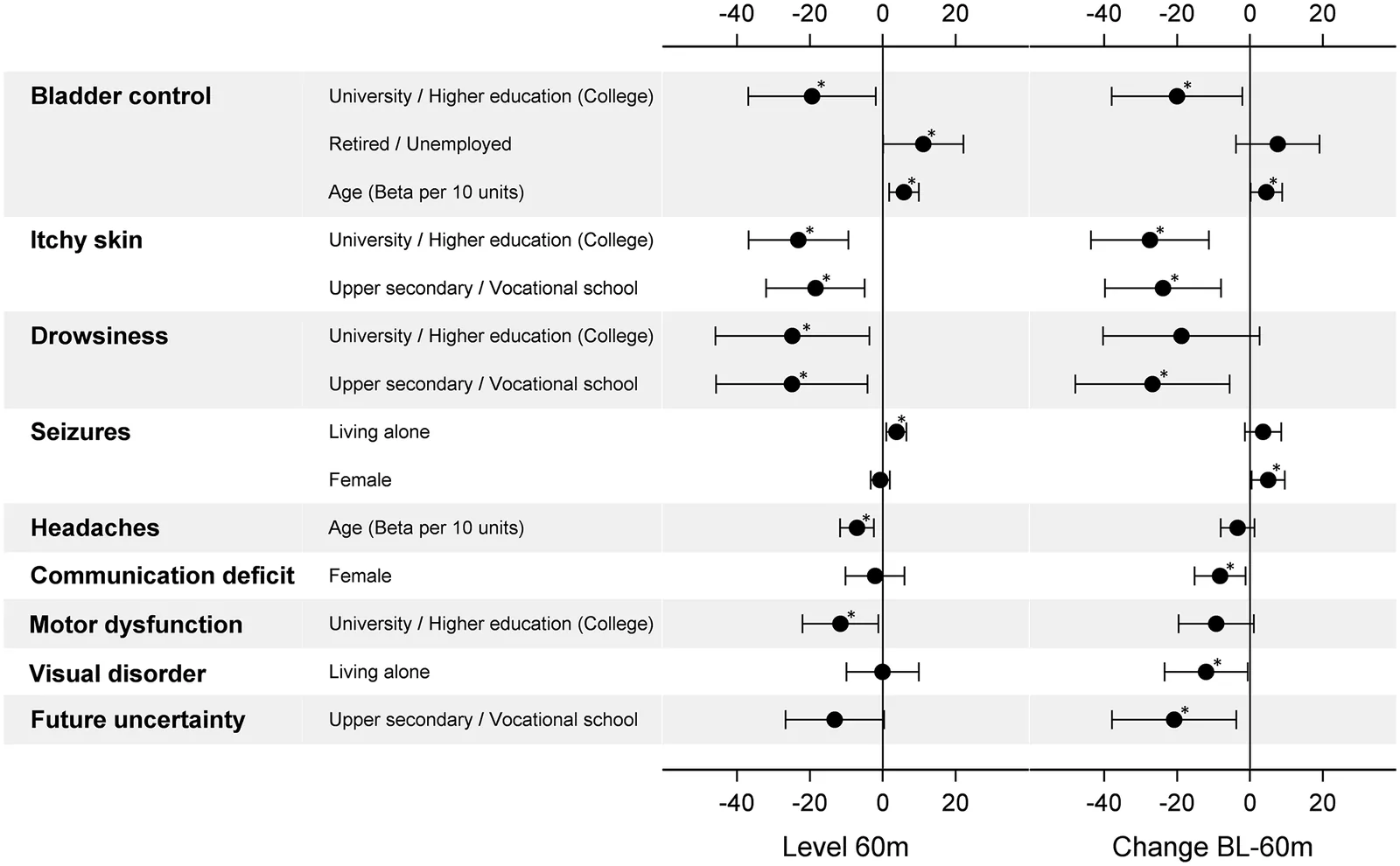
Univariable linear regression of selected QLQ-BN20 domain levels at 60 months (left) and changes from baseline to 60 months (right). Higher QLQ-BN20 scores indicate greater symptom burden, positive change indicates worsening and negative change indicates improvement. Beta coefficients and 95% CI are based on the original continuous variables. * *p* < 0.05. Only domains with at least one statistically significant association are displayed

In univariable analyses of baseline-to-60-month change (Fig. 4, Supplementary Table 3.1.1–4.11.2), older age was associated with greater deterioration in role functioning (β per 10 years − 6.90; *p* = 0.041) and social functioning (β per 10 years − 7.73; *p* = 0.0048). Being retired, on disability pension, unemployed or without an occupation was associated with greater declines in role functioning (β − 27.00; *p* = 0.0021) and social functioning (β − 15.49; *p* = 0.034). Higher education level was associated with more favourable change in social functioning (overall *p* = 0.021) and with selected QLQ-BN20 outcomes.

Change from baseline to five years did not differ between men and women across QLQ-C30 or QLQ-BN20 domains, and no investigated sociodemographic factors was associated with change in fatigue (all *p* ≥ 0.18) or insomnia (all *p* ≥ 0.33). Age-related differences in QLQ-BN20 change were most evident for bladder control, while most other domains showed no differences by age.

## Discussion

This longitudinal study provides one of the longest prospective evaluations of patient-reported outcomes after PBT in a predominantly meningioma cohort. The findings indicate a decline in participants’ quality of life over the five-year follow-up period, with worsening of several symptoms and functional domains. Specifically, fatigue and insomnia increased significantly along with cognitive functioning, suggesting an impact on patients’ everyday life. In contrast, changes in physical functioning were not clearly clinically meaningful, whereas emotional functioning improved to a statistically significant level, although its clinical relevance remains uncertain. Importantly, global health status remained relatively stable, suggesting that overall perceptions of health may be maintained despite changes in specific symptoms and functional domains. Fatigue and sleep disturbance may however be related to difficulties with concentration and memory, although this association was not examined here.

The present study adds a long-term patient-reported perspective on outcomes following PBT. However, without a photon-treated comparison group, the findings do not indicate whether patient-reported outcomes differ between PBT and conventional/photon radiotherapy. Photon-treated meningioma cohorts have reported persistent changes in long-term HRQoL impairment, while systematic reviews conclude that the contributions of the tumor and its treatment remain difficult to distinguish [3, 15]. Given the reduced dose to uninvolved tissues with PBT, potential benefits may be most relevant to late outcomes associated with exposure to normal brain and critical structures, including neurocognitive, endocrine, sensory or vascular effects [9–11]. However, current clinical evidence is insufficient to predict the direction or magnitude of differences for individual patient-reported symptoms. The present findings therefore describe outcomes after PBT but do not demonstrate effects by PBT.

No associations were observed between the investigated sociodemographic variables and longitudinal changes in HRQoL or symptoms. The observed changes in HRQoL and symptom burden may nevertheless reflect a combination of patient-, disease-, and treatment-related factors such as ageing, comorbidity, reduced physical activity, medication, endocrine effects, persistent tumor or surgery-related morbidity, and late treatment effects. Selective attrition over the follow-up period may also have contributed to the observed changes. Fatigue and sleep disturbance may also contribute to poorer self-reported concentration and memory [24, 25], although this association was not examined here. Given these multiple potential contributors, we consider the observed changes to reflect more than late effects of PBT alone.

In our study, declining cognitive functioning over time may reflect increasing perceived difficulties with concentration, and memory. Similar concerns have been reported previously [25] and may coexist with fatigue, sleep disturbance, seizures, and mood symptoms, with medication, all of which may impact cognitive functioning. The coexistence of global health status with deterioration in specific domains may partly reflect adaptation or response shift. This interpretation is supported by qualitative findings from long-term survivors within the same study cohort as the present study, who described acceptance, altered routines and reliance on family or partner support while living with persistent fatigue, cognitive difficulties and reduced autonomy [26]. Such adaptation may change how individuals perceive and rate their overall quality of life over time [27], even when symptoms and functional difficulties persist.

The increase in constipation may be more possibly related to factors such as medication, reduced activity, diet, ageing, comorbidity or endocrine dysfunction, none of which was measured adequately in this study, although a contribution from cranial treatment cannot be excluded. Financial difficulties remained low and statistically unchanged, which partly may be explained by Sweden´s predominantly tax-funded healthcare system and universal coverage [28]. Nevertheless, indirect costs, reduced work capacity and income effects can persist. The financial findings should therefore be interpreted within the Swedish healthcare context and may differ in healthcare systems where patients bear a greater share of treatment-related costs.

We found changes in drowsiness, motor dysfunction, weakness of legs and bladder control which highlight the relevance of long-term clinical assessment of mobility, rehabilitation needs, as well as urinary symptoms. Less favourable role and social functioning among older participants and those outside employment may further indicate differences in long-term functional and psychosocial rehabilitation, although these associations were exploratory and involved multiple outcomes. The cohort in this study comprised mainly menigioma patients and the remaining diagnostic groups were too small for reliable subtype comparisons. We suggest observed symptoms may be related to several factors, including tumor location, prior surgery, radiation exposure, ageing, and comorbidity.

### Strengths and limitations

Strengths include the prospective design with repeated assessments through five years and use of validated general and brain tumor–specific PRO instruments with clinically interpretable thresholds and MIDs, consistent with international guidance, SISAQOL principles [20–23, 29]. Reporting both absolute trajectories and change-from-baseline estimates with confidence intervals supports interpretation of the direction, magnitude and uncertainty of the findings.

Loss to follow-up at five years may have introduced selection bias, and the slightly higher educational level, more loss to follow up among male participants, and participants living alone among responders, may reduce generalizability. The absence of a photon-treated comparison group hinders direct comparative conclusions, and the observed outcomes may partly reflect underlying disease characteristics and treatment context rather than proton therapy itself. The lack of detailed data on dose and target volumes, medication, endocrine factors, and comorbidities further limits assessment of factors potentially contributing to long-term HRQoL.Cognitive functioning was assessed using two items, capturing relevant aspects of this domain while not fully reflecting its complexity. Sick-leave data were not collected, limiting a more comprehensive assessment of work capacity and indirect economic burden. The number of exploratory outcomes and univariable associations increase the potential for chance findings, which limits causal conclusions.

## Conclusions

Five-year outcomes after PBT in this predominantly meningioma cohort were mixed. Global health status was statistically unchanged, but the QLQ-C30 summary score declined modestly and clinically important fatigue and insomnia coexisted with smaller functional and brain tumor specific deteriorations. These findings support structured long-term symptom surveillance and rehabilitation. The absence of a photon-treated comparison group substantial attrition and limited clinical detail mean that the observed changes cannot be attributes specifically to PBT or to a particular tumor or treatment characteristic.

## Supplementary Information

Below is the link to the electronic supplementary material.

Supplementary Material 1

## Data Availability

No datasets were generated or analysed during the current study.
