# Supplementary material for: Five-year changes in health-related quality of life after proton therapy in a primarily meningioma cohort: a prospective study
Source: J Neurooncol. 2026 Aug 21;179(2):49. doi: 10.1007/s11060-026-05767-1 (PMC13498638; doi:10.1007/s11060-026-05767-1)
Supplement: Supplementary file 1 [file 11060_2026_5767_MOESM1_ESM.docx]

Tables and Figures

Supplement

Long-term health-related quality of life after proton therapy for benign brain tumors: a 5-year prospective cohort

# List of Tables

[**Table 1.1 Baseline**	4](#_Toc214286171)

[**Table 1.2 Baseline (only complete 60m)**	5](#_Toc214286172)

[**Table 2.1.1 EORTC QLQ-C30**	6](#_Toc214286173)

[**Table 2.1.2 Change in EORTC QLQ-C30**	7](#_Toc214286174)

[**Table 2.1.3 Model-Estimated Means (95% CI) for EORTC QLQ-C30 Subscales from the MMRM Analysis**	9](#_Toc214286175)

[**Table 2.1.4 Model-Estimated Difference from Baseline (95% CI) for EORTC QLQ-C30 Subscales from the MMRM Analysis**	10](#_Toc214286176)

[**Table 2.1.5 Model-Estimated Difference Baseline-60m between Men and Women (95% CI) from the MMRM Analysis**	11](#_Toc214286177)

[**Table 2.1.6 Model-Estimated Difference Baseline-60m between Age Categories (95% CI) from the MMRM Analysis**	12](#_Toc214286178)

[**Table 2.1.7 Model-Estimated Difference Baseline-60m between Education Categories (95% CI) from the MMRM Analysis**	13](#_Toc214286179)

[**Table 2.2.1 BN20**	14](#_Toc214286180)

[**Table 2.2.2 Change in BN20**	15](#_Toc214286181)

[**Table 2.2.3 Model-Estimated Means (95% CI) for BN20 from the MMRM Analysis**	17](#_Toc214286182)

[**Table 2.2.4 Model-Estimated Difference from Baseline (95% CI) for BN20 Subscales from the MMRM Analysis**	18](#_Toc214286183)

[**Table 2.2.5 Model-Estimated Difference Baseline-60m between Men and Women (95% CI) from the MMRM Analysis BN20**	19](#_Toc214286184)

[**Table 2.2.6 Model-Estimated Difference Baseline-60m between Age Categories (95% CI) from the MMRM Analysis**	20](#_Toc214286185)

[**Table 2.2.7 Model-Estimated Difference Baseline-60m between Education Categories (95% CI) from the MMRM Analysis**	21](#_Toc214286186)

[**Table 3.1.1 Univariable Linear Regression of Physical Functioning 60m**	22](#_Toc214286187)

[**Table 3.1.2 Univariable Linear Regression of Change in Physical Functioning (BL–60m)**	23](#_Toc214286188)

[**Table 3.2.1 Univariable Linear Regression of Role Functioning 60m**	24](#_Toc214286189)

[**Table 3.2.2 Univariable Linear Regression of Change in Role Functioning (BL–60m)**	25](#_Toc214286190)

[**Table 3.3.1 Univariable Linear Regression of Emotional Functioning 60m**	26](#_Toc214286191)

[**Table 3.3.2 Univariable Linear Regression of Change in Emotional Functioning (BL–60m)**	27](#_Toc214286192)

[**Table 3.4.1 Univariable Linear Regression of Cognitive Functioning 60m**	28](#_Toc214286193)

[**Table 3.4.2 Univariable Linear Regression of Change in Cognitive Functioning (BL–60m)**	29](#_Toc214286194)

[**Table 3.5.1 Univariable Linear Regression of Social Functioning 60m**	30](#_Toc214286195)

[**Table 3.5.2 Univariable Linear Regression of Change in Social Functioning (BL–60m)**	31](#_Toc214286196)

[**Table 3.6.1 Univariable Linear Regression of Global Health Status 60m**	32](#_Toc214286197)

[**Table 3.6.2 Univariable Linear Regression of Change in Global Health Status (BL–60m)**	33](#_Toc214286198)

[**Table 4.1.1 Univariable Linear Regression of Future uncertainty**	34](#_Toc214286199)

[**Table 4.1.2 Univariable Linear Regression in Change of Future uncertainty (BL-60m)**	35](#_Toc214286200)

[**Table 4.2.1 Univariable Linear Regression of Visual disorder**	36](#_Toc214286201)

[**Table 4.2.2 Univariable Linear Regression in Change of Visual disorder (BL-60m)**	37](#_Toc214286202)

[**Table 4.3.1 Univariable Linear Regression of Motor dysfunction**	38](#_Toc214286203)

[**Table 4.3.2 Univariable Linear Regression in Change of Motor dysfunction (BL-60m)**	39](#_Toc214286204)

[**Table 4.4.1 Univariable Linear Regression of Communication deficit**	40](#_Toc214286205)

[**Table 4.4.2 Univariable Linear Regression in Change of Communication deficit (BL-60m)**	41](#_Toc214286206)

[**Table 4.5.1 Univariable Linear Regression of Headaches**	42](#_Toc214286207)

[**Table 4.5.2 Univariable Linear Regression in Change of Headaches (BL-60m)**	43](#_Toc214286208)

[**Table 4.6.1 Univariable Linear Regression of Seizures**	44](#_Toc214286209)

[**Table 4.6.2 Univariable Linear Regression in Change of Seizures (BL-60m)**	45](#_Toc214286210)

[**Table 4.7.1 Univariable Linear Regression of Drowsiness**	46](#_Toc214286211)

[**Table 4.7.2 Univariable Linear Regression in Change of Drowsiness (BL-60m)**	47](#_Toc214286212)

[**Table 4.8.1 Univariable Linear Regression of Itchy skin**	48](#_Toc214286213)

[**Table 4.8.2 Univariable Linear Regression in Change of Itchy skin (BL-60m)**	49](#_Toc214286214)

[**Table 4.9.1 Univariable Linear Regression of Hair loss**	50](#_Toc214286215)

[**Table 4.9.2 Univariable Linear Regression in Change of Hair loss (BL-60m)**	51](#_Toc214286216)

[**Table 4.10.1 Univariable Linear Regression of Weakness of legs**	52](#_Toc214286217)

[**Table 4.10.2 Univariable Linear Regression in Change of Weakness of legs (BL-60m)**	53](#_Toc214286218)

[**Table 4.11.1 Univariable Linear Regression of Bladder control**	54](#_Toc214286219)

[**Table 4.11.2 Univariable Linear Regression in Change of Bladder control (BL-60m)**	55](#_Toc214286220)

# List of Figures

[**Figure 1 Kaplan-Meier**	57](#_Toc224113310)

**Table 1.1 Baseline**

| **Variable** | **All (n=126)** |
| --- | --- |
| **Age** | 55.3 (13.6) 56 (22; 80) n=126 |
| **Sex** |  |
| **Male** | 43 (34.1%) |
| **Female** | 83 (65.9%) |
| **Marital status** |  |
| **Married / Cohabiting (living with a partner)** | 90 (71.4%) |
| **Living alone (single household)** | 36 (28.6%) |
| **Employment status** |  |
| **Employed / Student** | 85 (67.5%) |
| **Retired / Disability pension / Unemployed / No occupation** | 41 (32.5%) |
| **Education level** |  |
| **Compulsory school** | 17 (13.6%) |
| **Upper secondary / Vocational school** | 60 (48.0%) |
| **University /Higher education (College)** | 48 (38.4%) |
| **ICD-10 code** |  |
| **D32.0** | 92 (73.0%) |
| **D32.1** | 1 (0.8%) |
| **D32.2** | 3 (2.4%) |
| **D32.9** | 10 (7.9%) |
| **D32.2** | 1 (0.8%) |
| **D33.0** | 3 (2.4%) |
| **D33.3** | 5 (4.0%) |
| **D33.9** | 2 (1.6%) |
| **D34.0** | 3 (2.4%) |
| **D35.2** | 3 (2.4%) |
| **D35.3** | 2 (1.6%) |
| **D44.3** | 1 (0.8%) |
| For categorical variables n (%) is presented. For continuous variables Mean (SD) / Median (Min; Max) / n= is presented. | |

**Table 1.2 Baseline (only complete 60m)**

| **Variable** | **All (n=77)** |
| --- | --- |
| **Age** | 56.5 (12.7) 57 (28; 74) n=77 |
| **Sex** |  |
| **Male** | 24 (31.2%) |
| **Female** | 53 (68.8%) |
| **Marital status** |  |
| **Married / Cohabiting (living with a partner)** | 59 (76.6%) |
| **Living alone (single household)** | 18 (23.4%) |
| **Employment status** |  |
| **Employed / Student** | 50 (64.9%) |
| **Retired / Disability pension / Unemployed / No occupation** | 27 (35.1%) |
| **Education level** |  |
| **Compulsory school** | 9 (11.8%) |
| **Upper secondary / Vocational school** | 36 (47.4%) |
| **University /Higher education (College)** | 31 (40.8%) |
| **ICD-10 code** |  |
| **D32.0** | 63 (81.8%) |
| **D32.1** | 1 (1.3%) |
| **D32.2** | 1 (1.3%) |
| **D32.9** | 4 (5.2%) |
| **D33.9** | 1 (1.3%) |
| **D34.0** | 2 (2.6%) |
| **D35.2** | 3 (3.9%) |
| **D35.3** | 2 (2.6%) |
| For categorical variables n (%) is presented. For continuous variables Mean (SD) / Median (Min; Max) / n= is presented. | |

**Table 1.3 Baseline (only without complete 60m)**

| **Variable** | **All (n=49)** |
| --- | --- |
| **Age** | 53.4 (14.8) 53 (22; 80) n=49 |
| **Sex** |  |
| **Male** | 19 (38.8%) |
| **Female** | 30 (61.2%) |
| **Marital status** |  |
| **Married / Cohabiting (living with a partner)** | 31 (63.3%) |
| **Living alone (single household)** | 18 (36.7%) |
| **Employment status** |  |
| **Employed / Student** | 35 (71.4%) |
| **Retired / Disability pension / Unemployed / No occupation** | 14 (28.6%) |
| **Education level** |  |
| **Compulsory school** | 8 (16.3%) |
| **Upper secondary / Vocational school** | 24 (49.0%) |
| **University /Higher education (College)** | 17 (34.7%) |
| **ICD code** |  |
| **D32.0** | 29 (59.2%) |
| **D32.2** | 2 (4.1%) |
| **D32.9** | 6 (12.2%) |
| **D32.2** | 1 (2.0%) |
| **D33.0** | 3 (6.1%) |
| **D33.3** | 5 (10.2%) |
| **D33.9** | 1 (2.0%) |
| **D34.0** | 1 (2.0%) |
| **D44.3** | 1 (2.0%) |
| For categorical variables n (%) is presented. For continuous variables Mean (SD) / Median (Min; Max) / n= is presented. | |

**Table 2.1.1 EORTC QLQ-C30**

| **Variable** | **Baseline (n=126)** | **Mid (n=126)** | **End (n=126)** | **1m (n=126)** | **3m (n=126)** | **6m (n=126)** | **9m (n=126)** | **12m (n=126)** | **24m (n=126)** | **36m (n=126)** | **60m (n=126)** |
| --- | --- | --- | --- | --- | --- | --- | --- | --- | --- | --- | --- |
| **Fatigue** | 29.4 (22.2) n=126 | 36.8 (25.6) n=121 | 42.0 (26.8) n=120 | 40.8 (26.4) n=122 | 39.2 (24.9) n=121 | 32.4 (23.1) n=113 | 33.4 (24.9) n=112 | 34.5 (24.6) n=103 | 33.9 (25.6) n=105 | 35.7 (24.6) n=92 | 38.1 (24.7) n=77 |
| **Nausea** | 3.97 (12.37) n=126 | 7.85 (16.26) n=121 | 7.78 (14.64) n=120 | 5.60 (11.86) n=122 | 8.26 (17.76) n=121 | 4.72 (12.88) n=113 | 3.87 (9.49) n=112 | 3.24 (9.35) n=103 | 4.13 (9.47) n=105 | 5.07 (12.29) n=92 | 4.33 (11.60) n=77 |
| **Pain** | 16.1 (23.1) n=126 | 20.1 (24.8) n=121 | 23.5 (27.5) n=120 | 20.9 (26.9) n=122 | 19.3 (25.3) n=121 | 18.7 (26.6) n=113 | 21.1 (26.2) n=112 | 19.6 (24.5) n=103 | 18.3 (24.7) n=105 | 20.8 (23.4) n=92 | 22.1 (27.9) n=77 |
| **Dyspnoea** | 15.5 (23.4) n=125 | 16.8 (24.0) n=121 | 21.9 (25.7) n=120 | 24.3 (29.1) n=122 | 24.0 (28.3) n=121 | 18.3 (21.4) n=113 | 16.7 (22.4) n=112 | 17.8 (21.3) n=103 | 17.5 (22.7) n=105 | 20.3 (22.6) n=92 | 20.3 (23.0) n=77 |
| **Insomnia** | 24.1 (29.7) n=126 | 30.0 (29.0) n=121 | 31.1 (31.7) n=120 | 23.2 (29.0) n=122 | 22.9 (30.1) n=121 | 22.4 (28.0) n=113 | 24.7 (28.6) n=112 | 23.6 (28.2) n=103 | 29.2 (30.6) n=105 | 31.5 (30.2) n=92 | 33.8 (28.9) n=77 |
| **Appetite** | 7.94 (17.09) n=126 | 12.7 (23.7) n=121 | 13.6 (22.2) n=120 | 9.92 (20.48) n=121 | 14.3 (23.9) n=121 | 8.55 (19.30) n=113 | 7.44 (18.83) n=112 | 8.41 (17.29) n=103 | 7.62 (18.64) n=105 | 7.61 (19.20) n=92 | 6.49 (16.26) n=77 |
| **Constipation** | 7.94 (20.84) n=126 | 8.82 (20.08) n=121 | 13.3 (25.0) n=120 | 7.38 (17.41) n=122 | 11.6 (21.8) n=121 | 8.26 (19.69) n=113 | 8.93 (21.45) n=112 | 8.74 (19.22) n=103 | 13.7 (25.6) n=105 | 14.5 (25.3) n=92 | 14.3 (25.0) n=77 |
| **Diarrhoea** | 4.76 (12.45) n=126 | 6.34 (14.47) n=121 | 6.94 (16.68) n=120 | 7.38 (17.41) n=122 | 7.99 (18.27) n=121 | 5.60 (14.02) n=113 | 4.17 (11.07) n=112 | 4.85 (14.32) n=103 | 6.03 (14.46) n=105 | 6.52 (16.57) n=92 | 8.23 (16.36) n=77 |
| **Financial difficulties** | 16.4 (25.9) n=126 | 15.6 (24.8) n=120 | 18.3 (28.0) n=120 | 19.9 (29.0) n=122 | 15.7 (26.2) n=121 | 13.0 (26.5) n=113 | 15.2 (27.2) n=112 | 15.2 (26.3) n=103 | 11.7 (24.5) n=105 | 10.1 (22.5) n=92 | 9.96 (22.98) n=77 |
| **Global Health Status** | 68.3 (19.0) n=126 | 66.1 (18.9) n=121 | 61.2 (22.0) n=120 | 62.8 (23.7) n=122 | 62.6 (20.9) n=121 | 65.4 (22.9) n=113 | 65.0 (23.2) n=112 | 62.5 (23.7) n=103 | 67.0 (21.3) n=105 | 62.6 (23.7) n=92 | 66.1 (20.9) n=77 |
| **Physical functioning** | 85.1 (17.5) n=126 | 83.9 (19.0) n=121 | 83.1 (18.7) n=120 | 82.2 (19.3) n=122 | 82.4 (16.8) n=121 | 84.7 (17.6) n=113 | 83.9 (20.4) n=112 | 84.5 (20.4) n=103 | 83.2 (20.6) n=105 | 83.6 (18.9) n=92 | 81.7 (18.4) n=77 |
| **Role functioning** | 67.6 (33.4) n=126 | 64.2 (34.1) n=121 | 60.1 (32.7) n=120 | 62.6 (31.7) n=122 | 66.1 (30.6) n=121 | 73.7 (31.9) n=113 | 67.9 (33.8) n=112 | 71.8 (32.0) n=103 | 72.5 (33.7) n=105 | 70.3 (32.3) n=92 | 69.0 (34.1) n=77 |
| **Emotional functioning** | 74.9 (22.3) n=126 | 83.7 (19.2) n=121 | 82.9 (21.9) n=120 | 80.7 (22.3) n=122 | 78.4 (21.7) n=121 | 78.8 (23.7) n=113 | 78.3 (24.3) n=112 | 77.8 (23.1) n=103 | 77.4 (25.4) n=105 | 76.9 (22.3) n=92 | 81.1 (18.5) n=77 |
| **Cognitive functioning** | 79.8 (20.9) n=126 | 80.0 (20.9) n=121 | 77.9 (21.9) n=120 | 74.9 (22.9) n=122 | 75.3 (22.0) n=121 | 76.0 (23.9) n=113 | 77.4 (23.1) n=112 | 76.1 (22.5) n=103 | 73.5 (24.4) n=105 | 75.0 (21.6) n=92 | 76.0 (21.5) n=77 |
| **Social functioning** | 77.5 (25.8) n=126 | 74.9 (25.7) n=121 | 76.0 (25.5) n=120 | 75.7 (26.9) n=122 | 78.5 (25.0) n=121 | 78.9 (26.0) n=113 | 79.6 (25.2) n=112 | 76.9 (28.8) n=103 | 78.7 (29.0) n=105 | 78.1 (27.7) n=92 | 79.9 (26.0) n=77 |
| For continuous variables Mean (SD) / n= is presented.   Effect size is estimated using the Cohen method (difference in mean/SD at first timepoint). | | | | | | | | | | | |

**Table 2.1.2 Change in EORTC QLQ-C30**

| **Variable** | **Change from Baseline to Mid** | **Change from Baseline to End** | **Change from Baseline to 1m** | **Change from Baseline to 3m** | **Change from Baseline to 6m** | **Change from Baseline to 9m** | **Change from Baseline to 12m** | **Change from Baseline to 24m** | **Change from Baseline to 36m** | **Change from Baseline to 60m** |
| --- | --- | --- | --- | --- | --- | --- | --- | --- | --- | --- |
| **Fatigue** | 7.25 (3.83; 10.75) n=121 p=<.0001 ES=0.33 | 12.7 (8.8; 16.7) n=120 p=<.0001 ES=0.58 | 11.1 (7.2; 15.1) n=122 p=<.0001 ES=0.50 | 9.09 (5.30; 12.87) n=121 p=<.0001 ES=0.41 | 2.36 (-1.59; 6.25) n=113 p=0.24 ES=0.11 | 4.37 (0.00; 8.89) n=112 p=0.055 ES=0.20 | 5.72 (1.48; 9.88) n=103 p=0.0081 ES=0.26 | 4.87 (0.65; 9.26) n=105 p=0.028 ES=0.22 | 6.76 (1.77; 11.87) n=92 p=0.0089 ES=0.32 | 10.7 (4.9; 16.4) n=77 p=0.0004 ES=0.51 |
| **Nausea** | 3.72 (1.47; 6.01) n=121 p=0.0016 ES=0.30 | 3.61 (1.21; 6.06) n=120 p=0.0037 ES=0.29 | 1.64 (-0.29; 3.57) n=122 p=0.096 ES=0.13 | 4.27 (1.30; 7.27) n=121 p=0.0055 ES=0.34 | 0.737 (-1.754; 3.175) n=113 p=0.56 ES=0.06 | 0.446 (-1.923; 2.830) n=112 p=0.72 ES=0.04 | -0.324 (-2.381; 1.736) n=103 p=0.76 ES=-0.03 | 0.635 (-1.812; 3.061) n=105 p=0.61 ES=0.06 | 2.54 (-0.34; 5.41) n=92 p=0.075 ES=0.34 | 1.52 (-1.22; 4.26) n=77 p=0.28 ES=0.19 |
| **Pain** | 3.58 (0.00; 7.14) n=121 p=0.048 ES=0.15 | 7.08 (2.65; 11.58) n=120 p=0.0022 ES=0.30 | 4.51 (0.53; 8.50) n=122 p=0.030 ES=0.19 | 2.89 (-0.98; 6.73) n=121 p=0.14 ES=0.12 | 2.65 (-1.77; 7.14) n=113 p=0.24 ES=0.11 | 5.65 (1.37; 9.94) n=112 p=0.010 ES=0.25 | 4.21 (-0.31; 8.65) n=103 p=0.063 ES=0.19 | 2.86 (-1.45; 7.19) n=105 p=0.20 ES=0.13 | 5.07 (0.40; 9.78) n=92 p=0.033 ES=0.23 | 7.14 (1.85; 12.37) n=77 p=0.0084 ES=0.32 |
| **Dyspnoea** | 0.833 (-2.924; 4.667) n=120 p=0.67 ES=0.04 | 6.44 (2.15; 10.77) n=119 p=0.0039 ES=0.28 | 8.82 (4.04; 13.73) n=121 p=0.0004 ES=0.37 | 8.06 (3.24; 12.87) n=120 p=0.0011 ES=0.34 | 2.38 (-2.38; 7.02) n=112 p=0.31 ES=0.10 | 0.901 (-3.448; 5.229) n=111 p=0.68 ES=0.04 | 2.29 (-2.27; 6.82) n=102 p=0.32 ES=0.09 | 1.92 (-3.47; 7.27) n=104 p=0.48 ES=0.08 | 5.86 (0.00; 11.38) n=91 p=0.038 ES=0.26 | 7.46 (1.01; 13.89) n=76 p=0.021 ES=0.34 |
| **Insomnia** | 4.96 (0.00; 9.84) n=121 p=0.046 ES=0.17 | 6.11 (0.54; 11.86) n=120 p=0.037 ES=0.20 | -1.09 (-6.21; 4.10) n=122 p=0.68 ES=-0.04 | -1.65 (-6.80; 3.51) n=121 p=0.53 ES=-0.06 | -1.77 (-6.88; 3.27) n=113 p=0.49 ES=-0.06 | 0.595 (-4.242; 5.556) n=112 p=0.81 ES=0.02 | -0.000 (-5.926; 6.061) n=103 p=1.00 ES=-0.00 | 6.03 (0.00; 12.18) n=105 p=0.056 ES=0.20 | 6.16 (-0.69; 12.77) n=92 p=0.071 ES=0.21 | 10.4 (2.8; 17.9) n=77 p=0.0072 ES=0.38 |
| **Appetite** | 4.68 (1.00; 8.47) n=121 p=0.017 ES=0.27 | 5.56 (1.39; 9.72) n=120 p=0.0093 ES=0.32 | 1.93 (-1.69; 5.56) n=121 p=0.30 ES=0.11 | 6.34 (2.15; 10.67) n=121 p=0.0039 ES=0.37 | 0.295 (-3.571; 4.094) n=113 p=0.88 ES=0.02 | 0.000 (-4.242; 4.167) n=112 p=1.00 ES=0.00 | 0.971 (-2.721; 4.667) n=103 p=0.60 ES=0.06 | 0.000 (-4.242; 4.242) n=105 p=1.00 ES=0.00 | 0.725 (-3.623; 5.128) n=92 p=0.75 ES=0.05 | 0.000 (-4.762; 4.762) n=77 p=1.00 ES=0.00 |
| **Constipation** | 0.551 (-3.226; 4.301) n=121 p=0.77 ES=0.03 | 5.00 (0.56; 9.37) n=120 p=0.028 ES=0.23 | -0.820 (-4.167; 2.469) n=122 p=0.62 ES=-0.04 | 3.31 (-0.91; 7.47) n=121 p=0.12 ES=0.16 | -0.590 (-4.255; 3.125) n=113 p=0.75 ES=-0.03 | 0.893 (-3.125; 4.918) n=112 p=0.66 ES=0.04 | 1.94 (-2.78; 6.67) n=103 p=0.42 ES=0.11 | 5.71 (1.36; 10.00) n=105 p=0.010 ES=0.29 | 6.88 (1.96; 11.85) n=92 p=0.0071 ES=0.35 | 8.23 (2.02; 14.41) n=77 p=0.0095 ES=0.44 |
| **Diarrhoea** | 1.65 (-1.54; 4.84) n=121 p=0.31 ES=0.13 | 2.22 (-1.13; 5.46) n=120 p=0.18 ES=0.18 | 2.46 (-1.43; 6.32) n=122 p=0.21 ES=0.19 | 3.03 (-0.54; 6.52) n=121 p=0.086 ES=0.24 | 0.295 (-2.516; 3.086) n=113 p=0.84 ES=0.02 | -0.595 (-2.778; 1.639) n=112 p=0.60 ES=-0.05 | -0.324 (-3.101; 2.516) n=103 p=0.82 ES=-0.02 | 0.952 (-2.174; 4.082) n=105 p=0.55 ES=0.07 | 1.45 (-1.67; 4.67) n=92 p=0.37 ES=0.11 | 3.03 (0.00; 6.50) n=77 p=0.090 ES=0.23 |
| **Financial difficulties** | -0.833 (-4.301; 2.604) n=120 p=0.63 ES=-0.03 | 1.94 (-1.52; 5.36) n=120 p=0.26 ES=0.07 | 3.55 (-0.56; 7.60) n=122 p=0.085 ES=0.14 | -1.10 (-5.36; 3.09) n=121 p=0.61 ES=-0.04 | -4.13 (-8.47; -0.00) n=113 p=0.061 ES=-0.16 | -0.893 (-5.263; 3.448) n=112 p=0.69 ES=-0.04 | -0.647 (-4.762; 3.509) n=103 p=0.76 ES=-0.03 | -3.81 (-7.88; -0.00) n=105 p=0.070 ES=-0.15 | -4.71 (-9.52; -0.00) n=92 p=0.057 ES=-0.19 | -2.60 (-8.33; 2.96) n=77 p=0.37 ES=-0.12 |
| **Global Health Status** | -2.20 (-5.19; 0.76) n=121 p=0.15 ES=-0.12 | -7.22 (-10.96; -3.57) n=120 p=0.0002 ES=-0.39 | -5.05 (-8.93; -1.19) n=122 p=0.011 ES=-0.27 | -5.51 (-9.13; -1.92) n=121 p=0.0030 ES=-0.30 | -1.99 (-5.90; 1.96) n=113 p=0.32 ES=-0.11 | -2.90 (-7.08; 1.21) n=112 p=0.17 ES=-0.16 | -4.94 (-9.17; -0.79) n=103 p=0.021 ES=-0.27 | -0.556 (-4.885; 3.758) n=105 p=0.80 ES=-0.03 | -4.98 (-9.28; -0.68) n=92 p=0.023 ES=-0.27 | -1.84 (-6.37; 2.78) n=77 p=0.43 ES=-0.10 |
| **Physical functioning** | -1.10 (-3.15; 0.90) n=121 p=0.29 ES=-0.06 | -2.17 (-4.67; 0.32) n=120 p=0.088 ES=-0.12 | -3.03 (-5.42; -0.67) n=122 p=0.012 ES=-0.17 | -2.37 (-4.37; -0.37) n=121 p=0.021 ES=-0.13 | -0.177 (-2.099; 1.739) n=113 p=0.86 ES=-0.01 | -1.07 (-3.85; 1.70) n=112 p=0.44 ES=-0.06 | -0.583 (-3.457; 2.313) n=103 p=0.69 ES=-0.03 | -1.65 (-4.71; 1.36) n=105 p=0.29 ES=-0.09 | -2.75 (-6.02; 0.45) n=92 p=0.095 ES=-0.17 | -5.02 (-8.25; -1.75) n=77 p=0.0031 ES=-0.34 |
| **Role functioning** | -3.31 (-8.61; 2.04) n=121 p=0.23 ES=-0.10 | -7.50 (-13.84; -1.17) n=120 p=0.019 ES=-0.22 | -4.92 (-10.54; 0.76) n=122 p=0.085 ES=-0.15 | -1.52 (-7.29; 4.25) n=121 p=0.60 ES=-0.05 | 6.93 (0.96; 12.89) n=113 p=0.023 ES=0.21 | 0.446 (-6.433; 7.407) n=112 p=0.90 ES=0.01 | 4.69 (-2.30; 11.73) n=103 p=0.18 ES=0.14 | 4.60 (-2.71; 11.81) n=105 p=0.21 ES=0.13 | 2.54 (-5.70; 10.76) n=92 p=0.54 ES=0.08 | 0.866 (-7.609; 9.524) n=77 p=0.84 ES=0.03 |
| **Emotional functioning** | 9.78 (6.42; 13.11) n=121 p=<.0001 ES=0.44 | 9.03 (5.56; 12.43) n=120 p=<.0001 ES=0.40 | 5.94 (2.47; 9.44) n=122 p=0.0010 ES=0.27 | 4.06 (0.66; 7.49) n=121 p=0.021 ES=0.18 | 4.57 (0.57; 8.61) n=113 p=0.026 ES=0.20 | 3.57 (-0.58; 7.69) n=112 p=0.090 ES=0.16 | 2.59 (-1.67; 6.94) n=103 p=0.24 ES=0.12 | 3.10 (-1.17; 7.37) n=105 p=0.15 ES=0.14 | 1.72 (-3.15; 6.62) n=92 p=0.48 ES=0.08 | 4.98 (0.20; 9.87) n=77 p=0.046 ES=0.25 |
| **Cognitive functioning** | 0.275 (-2.968; 3.390) n=121 p=0.86 ES=0.01 | -2.08 (-5.45; 1.32) n=120 p=0.23 ES=-0.10 | -5.19 (-8.91; -1.44) n=122 p=0.0073 ES=-0.25 | -3.86 (-7.14; -0.55) n=121 p=0.023 ES=-0.18 | -3.69 (-7.55; 0.27) n=113 p=0.061 ES=-0.18 | -2.68 (-6.48; 1.11) n=112 p=0.16 ES=-0.13 | -3.07 (-6.86; 0.71) n=103 p=0.11 ES=-0.14 | -5.71 (-9.88; -1.54) n=105 p=0.0080 ES=-0.27 | -5.25 (-10.26; -0.35) n=92 p=0.039 ES=-0.25 | -5.41 (-10.29; -0.46) n=77 p=0.032 ES=-0.27 |
| **Social functioning** | -2.07 (-5.91; 1.79) n=121 p=0.29 ES=-0.08 | -0.833 (-4.762; 3.145) n=120 p=0.68 ES=-0.03 | -2.19 (-6.86; 2.42) n=122 p=0.36 ES=-0.09 | 1.79 (-2.42; 6.03) n=121 p=0.40 ES=0.07 | 1.92 (-2.59; 6.41) n=113 p=0.40 ES=0.08 | 1.49 (-3.29; 6.29) n=112 p=0.54 ES=0.06 | -0.324 (-5.903; 5.247) n=103 p=0.91 ES=-0.01 | 0.635 (-4.885; 6.090) n=105 p=0.82 ES=0.03 | -0.543 (-7.018; 5.797) n=92 p=0.86 ES=-0.02 | -0.433 (-7.447; 6.579) n=77 p=0.90 ES=-0.02 |
| For continuous variables Mean (SD) is presented. For change in continuous variables Mean (95% CI for Mean using the inversion of Fisher´s non-parametric permutation test) / n= is presented.  For comparison over time, the t-test was used for continuous variables. Effect size is estimated using the Cohen method (difference in mean/SD at first timepoint). | | | | | | | | | | |

**Table 2.1.3 Model-Estimated Means (95% CI) for EORTC QLQ-C30 Subscales from the** **MMRM Analysis**

|  | **Visit** | | | | | | | | | | |
| --- | --- | --- | --- | --- | --- | --- | --- | --- | --- | --- | --- |
|  | **Baseline** | **Mid** | **End** | **1m** | **3m** | **6m** | **9m** | **12m** | **24m** | **36m** | **60m** |
| Appetite | 7.8 (4.6, 11.0) | 12.5 (9.2, 15.7) | 13.4 (10.2, 16.7) | 9.5 (6.3, 12.8) | 14.0 (10.7, 17.3) | 8.9 (5.5, 12.3) | 7.8 (4.4, 11.2) | 9.0 (5.5, 12.6) | 7.6 (4.1, 11.1) | 8.2 (4.5, 11.9) | 7.4 (3.5, 11.3) |
| Cognitive functioning | 79.8 (76.8, 82.8) | 80.1 (77.0, 83.2) | 77.7 (74.6, 80.8) | 74.8 (71.8, 77.9) | 75.8 (72.7, 78.9) | 75.5 (72.3, 78.7) | 76.5 (73.3, 79.7) | 75.7 (72.4, 79.0) | 73.5 (70.2, 76.8) | 74.0 (70.6, 77.5) | 73.8 (70.2, 77.4) |
| Constipation | 7.9 (4.6, 11.2) | 8.7 (5.3, 12.0) | 13.2 (9.8, 16.5) | 7.1 (3.7, 10.4) | 11.4 (8.1, 14.8) | 8.6 (5.2, 12.1) | 9.2 (5.7, 12.6) | 9.7 (6.1, 13.3) | 13.3 (9.8, 16.9) | 14.4 (10.6, 18.1) | 15.9 (12.0, 19.9) |
| Diarrhoea | 4.8 (2.4, 7.3) | 6.4 (3.9, 8.9) | 6.9 (4.4, 9.4) | 7.3 (4.8, 9.8) | 8.2 (5.7, 10.7) | 5.7 (3.1, 8.3) | 4.2 (1.6, 6.8) | 4.7 (2.0, 7.4) | 6.0 (3.3, 8.7) | 6.3 (3.5, 9.2) | 7.7 (4.6, 10.8) |
| Dyspnoea | 15.4 (11.8, 19.1) | 16.4 (12.7, 20.1) | 21.9 (18.2, 25.6) | 24.6 (20.9, 28.2) | 23.7 (20.0, 27.4) | 18.7 (14.9, 22.5) | 16.7 (12.9, 20.6) | 17.6 (13.6, 21.6) | 17.4 (13.5, 21.4) | 21.4 (17.2, 25.5) | 21.4 (17.0, 25.7) |
| Emotional functioning | 74.7 (71.7, 77.7) | 84.2 (81.1, 87.3) | 83.3 (80.2, 86.4) | 80.6 (77.6, 83.7) | 78.6 (75.6, 81.7) | 78.6 (75.4, 81.7) | 77.8 (74.7, 81.0) | 77.2 (74.0, 80.5) | 77.2 (74.0, 80.5) | 76.5 (73.2, 79.9) | 79.0 (75.5, 82.4) |
| Fatigue | 29.3 (25.9, 32.7) | 36.6 (33.2, 40.1) | 42.0 (38.5, 45.4) | 40.5 (37.1, 44.0) | 38.8 (35.4, 42.3) | 32.3 (28.8, 35.8) | 33.5 (30.0, 37.1) | 34.9 (31.2, 38.6) | 33.8 (30.1, 37.4) | 36.9 (33.1, 40.7) | 40.4 (36.5, 44.4) |
| Financial difficulties | 16.1 (12.8, 19.4) | 15.2 (11.8, 18.6) | 17.8 (14.5, 21.2) | 19.6 (16.2, 22.9) | 15.3 (11.9, 18.7) | 12.7 (9.2, 16.1) | 15.4 (11.9, 18.9) | 15.1 (11.5, 18.7) | 11.6 (8.0, 15.2) | 9.8 (6.1, 13.6) | 12.7 (8.7, 16.6) |
| Global Health Status | 68.0 (64.9, 71.1) | 66.0 (62.8, 69.2) | 61.1 (57.9, 64.3) | 63.0 (59.8, 66.1) | 62.8 (59.6, 65.9) | 65.7 (62.5, 69.0) | 65.1 (61.8, 68.4) | 62.9 (59.5, 66.3) | 67.6 (64.2, 71.0) | 62.6 (59.1, 66.2) | 65.8 (62.1, 69.5) |
| Insomnia | 24.2 (19.9, 28.5) | 29.6 (25.2, 33.9) | 31.0 (26.7, 35.4) | 23.2 (18.9, 27.5) | 22.6 (18.3, 26.9) | 23.0 (18.6, 27.5) | 25.1 (20.6, 29.6) | 24.3 (19.6, 28.9) | 29.9 (25.3, 34.5) | 31.4 (26.5, 36.2) | 34.8 (29.7, 39.9) |
| Nausea | 3.8 (1.8, 5.8) | 7.6 (5.6, 9.6) | 7.5 (5.5, 9.5) | 5.4 (3.4, 7.4) | 8.1 (6.1, 10.0) | 4.6 (2.6, 6.7) | 4.2 (2.1, 6.3) | 3.3 (1.2, 5.5) | 4.2 (2.0, 6.3) | 5.5 (3.3, 7.8) | 5.1 (2.7, 7.5) |
| Pain | 16.0 (12.6, 19.4) | 19.6 (16.1, 23.1) | 22.9 (19.5, 26.4) | 20.7 (17.3, 24.2) | 18.7 (15.3, 22.2) | 19.0 (15.4, 22.5) | 21.6 (18.0, 25.2) | 19.7 (16.0, 23.4) | 18.4 (14.8, 22.1) | 21.7 (17.9, 25.5) | 24.1 (20.1, 28.1) |
| Physical functioning | 85.2 (83.1, 87.3) | 84.1 (81.9, 86.2) | 83.0 (80.9, 85.2) | 82.2 (80.1, 84.4) | 82.8 (80.7, 85.0) | 84.8 (82.6, 87.0) | 83.9 (81.7, 86.1) | 84.3 (82.0, 86.5) | 83.5 (81.3, 85.8) | 81.7 (79.3, 84.0) | 79.5 (77.1, 82.0) |
| QLQ-C30 Summary Score | 82.7 (80.9, 84.5) | 80.8 (79.0, 82.7) | 78.6 (76.8, 80.4) | 79.8 (78.0, 81.6) | 79.8 (77.9, 81.6) | 82.3 (80.5, 84.2) | 81.6 (79.7, 83.5) | 81.6 (79.7, 83.6) | 81.1 (79.2, 83.1) | 79.4 (77.4, 81.4) | 78.3 (76.2, 80.3) |
| Role functioning | 67.6 (62.7, 72.4) | 64.2 (59.3, 69.2) | 60.2 (55.3, 65.1) | 62.5 (57.6, 67.4) | 66.2 (61.3, 71.1) | 74.1 (69.1, 79.1) | 67.8 (62.8, 72.9) | 71.8 (66.7, 77.0) | 72.7 (67.5, 77.9) | 69.5 (64.1, 74.9) | 66.8 (61.3, 72.4) |
| Social functioning | 77.6 (73.8, 81.4) | 75.1 (71.3, 79.0) | 76.1 (72.3, 80.0) | 75.8 (71.9, 79.6) | 78.9 (75.1, 82.8) | 79.2 (75.3, 83.2) | 79.2 (75.2, 83.1) | 77.1 (73.0, 81.2) | 78.8 (74.7, 82.9) | 76.9 (72.7, 81.2) | 76.4 (72.0, 80.8) |

**Table 2.1.4 Model-Estimated Difference from Baseline (95% CI) for EORTC QLQ-C30 Subscales from the MMRM Analysis**

|  | **Visit** | | | | | | | | | |
| --- | --- | --- | --- | --- | --- | --- | --- | --- | --- | --- |
|  | **Mid** | **End** | **1m** | **3m** | **6m** | **9m** | **12m** | **24m** | **36m** | **60m** |
| Appetite | 4.6 (0.0, 9.2) p 0.048 | 5.6 (1.0, 10.2) p 0.017 | 1.7 (-2.9, 6.3) p 0.46 | 6.2 (1.6, 10.8) p 0.008 | 1.1 (-3.6, 5.7) p 0.65 | -0.0 (-4.7, 4.6) p 0.99 | 1.2 (-3.5, 5.9) p 0.61 | -0.3 (-4.8, 4.3) p 0.91 | 0.4 (-4.1, 4.9) p 0.86 | -0.4 (-4.4, 3.7) p 0.85 |
| Cognitive functioning | 0.3 (-4.1, 4.6) p 0.90 | -2.1 (-6.4, 2.3) p 0.35 | -5.0 (-9.3, -0.6) p 0.025 | -4.0 (-8.3, 0.4) p 0.072 | -4.3 (-8.6, 0.1) p 0.053 | -3.3 (-7.6, 1.1) p 0.14 | -4.1 (-8.4, 0.2) p 0.064 | -6.3 (-10.4, -2.1) p 0.003 | -5.7 (-9.7, -1.8) p 0.004 | -6.0 (-9.5, -2.6) p <.001 |
| Constipation | 0.7 (-4.0, 5.4) p 0.76 | 5.2 (0.5, 9.9) p 0.029 | -0.8 (-5.5, 3.8) p 0.72 | 3.5 (-1.2, 8.2) p 0.14 | 0.7 (-4.0, 5.4) p 0.77 | 1.2 (-3.5, 6.0) p 0.61 | 1.8 (-3.0, 6.5) p 0.47 | 5.4 (0.8, 10.0) p 0.021 | 6.4 (2.0, 10.9) p 0.005 | 8.0 (4.0, 12.0) p <.001 |
| Diarrhoea | 1.6 (-2.0, 5.1) p 0.38 | 2.1 (-1.5, 5.6) p 0.25 | 2.5 (-1.0, 6.0) p 0.17 | 3.4 (-0.2, 6.9) p 0.063 | 0.9 (-2.7, 4.5) p 0.64 | -0.7 (-4.3, 2.9) p 0.72 | -0.1 (-3.8, 3.5) p 0.95 | 1.2 (-2.4, 4.8) p 0.52 | 1.5 (-2.1, 5.1) p 0.42 | 2.9 (-0.5, 6.2) p 0.094 |
| Dyspnoea | 1.0 (-4.2, 6.1) p 0.72 | 6.5 (1.3, 11.7) p 0.015 | 9.1 (4.0, 14.3) p <.001 | 8.3 (3.1, 13.4) p 0.002 | 3.3 (-2.0, 8.5) p 0.22 | 1.3 (-3.9, 6.5) p 0.62 | 2.2 (-3.1, 7.4) p 0.41 | 2.0 (-3.1, 7.0) p 0.44 | 6.0 (1.1, 10.9) p 0.017 | 5.9 (1.6, 10.3) p 0.008 |
| Emotional functioning | 9.5 (5.2, 13.8) p <.001 | 8.6 (4.3, 12.9) p <.001 | 5.9 (1.7, 10.2) p 0.006 | 3.9 (-0.3, 8.2) p 0.070 | 3.9 (-0.4, 8.1) p 0.073 | 3.1 (-1.1, 7.3) p 0.14 | 2.5 (-1.6, 6.6) p 0.23 | 2.5 (-1.4, 6.4) p 0.21 | 1.8 (-1.8, 5.5) p 0.33 | 4.3 (1.1, 7.4) p 0.008 |
| Fatigue | 7.3 (2.5, 12.1) p 0.003 | 12.7 (7.9, 17.5) p <.001 | 11.2 (6.5, 16.0) p <.001 | 9.5 (4.7, 14.3) p <.001 | 3.0 (-1.8, 7.8) p 0.22 | 4.2 (-0.5, 9.0) p 0.081 | 5.6 (0.9, 10.3) p 0.020 | 4.5 (-0.0, 9.0) p 0.052 | 7.6 (3.3, 11.8) p <.001 | 11.1 (7.4, 14.8) p <.001 |
| Financial difficulties | -1.0 (-5.7, 3.8) p 0.69 | 1.7 (-3.0, 6.5) p 0.47 | 3.5 (-1.2, 8.2) p 0.15 | -0.8 (-5.5, 3.9) p 0.73 | -3.4 (-8.2, 1.3) p 0.15 | -0.7 (-5.4, 4.1) p 0.78 | -1.0 (-5.8, 3.7) p 0.66 | -4.5 (-9.1, 0.0) p 0.051 | -6.3 (-10.6, -1.9) p 0.005 | -3.4 (-7.3, 0.4) p 0.079 |
| Global Health Status | -2.0 (-6.5, 2.4) p 0.38 | -6.9 (-11.4, -2.4) p 0.002 | -5.1 (-9.5, -0.6) p 0.026 | -5.3 (-9.7, -0.8) p 0.020 | -2.3 (-6.8, 2.2) p 0.31 | -2.9 (-7.4, 1.5) p 0.20 | -5.1 (-9.5, -0.7) p 0.023 | -0.4 (-4.7, 3.8) p 0.84 | -5.4 (-9.4, -1.4) p 0.009 | -2.2 (-5.8, 1.3) p 0.22 |
| Insomnia | 5.4 (-0.7, 11.4) p 0.084 | 6.8 (0.7, 12.9) p 0.028 | -1.0 (-7.1, 5.0) p 0.74 | -1.6 (-7.6, 4.5) p 0.61 | -1.2 (-7.3, 5.0) p 0.71 | 0.9 (-5.2, 7.0) p 0.77 | 0.1 (-6.1, 6.2) p 0.99 | 5.7 (-0.3, 11.7) p 0.061 | 7.2 (1.4, 13.0) p 0.015 | 10.6 (5.4, 15.8) p <.001 |
| Nausea | 3.8 (1.0, 6.6) p 0.008 | 3.7 (0.9, 6.5) p 0.009 | 1.6 (-1.2, 4.4) p 0.26 | 4.3 (1.5, 7.0) p 0.003 | 0.8 (-2.0, 3.7) p 0.56 | 0.4 (-2.4, 3.2) p 0.78 | -0.5 (-3.3, 2.4) p 0.75 | 0.4 (-2.5, 3.2) p 0.80 | 1.7 (-1.1, 4.5) p 0.23 | 1.3 (-1.3, 3.9) p 0.32 |
| Pain | 3.6 (-1.3, 8.4) p 0.15 | 6.9 (2.1, 11.8) p 0.005 | 4.7 (-0.1, 9.6) p 0.056 | 2.7 (-2.1, 7.5) p 0.27 | 3.0 (-1.9, 7.8) p 0.23 | 5.6 (0.8, 10.4) p 0.022 | 3.7 (-1.1, 8.4) p 0.13 | 2.4 (-2.1, 7.0) p 0.29 | 5.7 (1.4, 10.0) p 0.009 | 8.1 (4.4, 11.8) p <.001 |
| Physical functioning | -1.2 (-4.2, 1.8) p 0.45 | -2.2 (-5.2, 0.8) p 0.15 | -3.0 (-5.9, 0.0) p 0.051 | -2.4 (-5.3, 0.6) p 0.11 | -0.4 (-3.4, 2.6) p 0.80 | -1.3 (-4.3, 1.6) p 0.38 | -1.0 (-3.8, 1.9) p 0.52 | -1.7 (-4.4, 1.1) p 0.23 | -3.6 (-6.1, -1.0) p 0.007 | -5.7 (-7.9, -3.5) p <.001 |
| QLQ-C30 Summary Score | -1.8 (-4.4, 0.7) p 0.16 | -4.1 (-6.6, -1.5) p 0.002 | -2.9 (-5.4, -0.3) p 0.026 | -2.9 (-5.4, -0.4) p 0.024 | -0.3 (-2.8, 2.1) p 0.79 | -1.0 (-3.5, 1.4) p 0.40 | -1.0 (-3.4, 1.3) p 0.39 | -1.6 (-3.8, 0.7) p 0.17 | -3.3 (-5.4, -1.2) p 0.002 | -4.4 (-6.1, -2.7) p <.001 |
| Role functioning | -3.3 (-10.2, 3.5) p 0.34 | -7.3 (-14.2, -0.5) p 0.036 | -5.1 (-11.9, 1.7) p 0.14 | -1.3 (-8.1, 5.4) p 0.70 | 6.6 (-0.2, 13.3) p 0.058 | 0.3 (-6.4, 7.0) p 0.94 | 4.3 (-2.3, 10.9) p 0.21 | 5.2 (-1.2, 11.5) p 0.11 | 1.9 (-4.0, 7.9) p 0.52 | -0.7 (-5.8, 4.4) p 0.78 |
| Social functioning | -2.5 (-7.9, 2.9) p 0.37 | -1.4 (-6.8, 3.9) p 0.60 | -1.8 (-7.2, 3.5) p 0.50 | 1.3 (-4.0, 6.7) p 0.62 | 1.6 (-3.8, 7.0) p 0.56 | 1.6 (-3.8, 6.9) p 0.57 | -0.4 (-5.8, 4.9) p 0.87 | 1.2 (-3.9, 6.2) p 0.65 | -0.6 (-5.5, 4.2) p 0.79 | -1.2 (-5.4, 3.0) p 0.58 |

**Table 2.1.5 Model-Estimated Difference Baseline-60m between Men and Women (95% CI) from the MMRM Analysis**

| **Variable** | **Change Men** | **Change Women** | **Change Difference** | **p-value** |
| --- | --- | --- | --- | --- |
| Cognitive functioning | -8.2 (-15.1, -1.3) | -6.2 (-10.9, -1.5) | 2.0 (-6.3, 10.4) | 0.64 |
| Emotional functioning | 3.1 (-3.4, 9.5) | 3.2 (-1.3, 7.6) | 0.1 (-7.8, 8.0) | 0.98 |
| Global Health Status | 0.5 (-6.6, 7.5) | -5.6 (-10.4, -0.7) | -6.0 (-14.6, 2.5) | 0.17 |
| Physical functioning | -5.4 (-10.4, -0.4) | -6.7 (-10.1, -3.2) | -1.3 (-7.4, 4.8) | 0.68 |
| Role functioning | -0.5 (-11.4, 10.4) | -2.1 (-9.6, 5.4) | -1.6 (-14.8, 11.6) | 0.81 |
| Social functioning | -2.1 (-10.7, 6.5) | -0.6 (-6.5, 5.3) | 1.5 (-9.0, 11.9) | 0.78 |
| Appetite | -4.9 (-12.9, 3.1) | 2.3 (-3.2, 7.8) | 7.3 (-2.4, 17.0) | 0.14 |
| Constipation | 4.3 (-3.7, 12.4) | 10.4 (4.9, 15.9) | 6.0 (-3.7, 15.8) | 0.22 |
| Diarrhoea | 1.1 (-5.3, 7.5) | 4.0 (-0.4, 8.4) | 2.9 (-4.9, 10.6) | 0.47 |
| Dyspnoea | 4.0 (-4.8, 12.7) | 8.2 (2.1, 14.2) | 4.2 (-6.4, 14.8) | 0.44 |
| Fatigue | 10.7 (3.0, 18.3) | 12.5 (7.3, 17.8) | 1.9 (-7.4, 11.2) | 0.69 |
| Financial difficulties | -3.2 (-11.2, 4.7) | -3.7 (-9.1, 1.8) | -0.4 (-10.1, 9.2) | 0.93 |
| Nausea | -1.6 (-6.4, 3.1) | 3.1 (-0.1, 6.4) | 4.8 (-1.0, 10.6) | 0.11 |
| Pain | 5.1 (-2.5, 12.7) | 10.0 (4.8, 15.2) | 4.9 (-4.3, 14.1) | 0.30 |
| Insomnia | 10.4 (0.4, 20.3) | 12.3 (5.5, 19.2) | 2.0 (-10.1, 14.1) | 0.75 |
| QLQ-C30 Summary Score | -3.2 (-7.0, 0.6) | -5.9 (-8.5, -3.2) | -2.6 (-7.3, 2.0) | 0.26 |

**Table 2.1.6 Model-Estimated Difference Baseline-60m between Age Categories (95% CI) from the MMRM Analysis**

| **Variable** | **Change <60** | **Change >=60** | **Change Difference** | **p-value** |
| --- | --- | --- | --- | --- |
| Cognitive functioning | -6.3 (-11.7, -1.0) | -7.2 (-13.0, -1.4) | -0.9 (-8.7, 7.0) | 0.83 |
| Emotional functioning | 4.2 (-0.8, 9.2) | 2.0 (-3.3, 7.4) | -2.2 (-9.5, 5.2) | 0.56 |
| Global Health Status | -2.9 (-8.3, 2.6) | -4.3 (-10.2, 1.5) | -1.5 (-9.5, 6.6) | 0.72 |
| Physical functioning | -4.7 (-8.5, -0.8) | -7.9 (-12.1, -3.8) | -3.2 (-8.9, 2.4) | 0.26 |
| Role functioning | 4.6 (-3.8, 13.0) | -8.5 (-17.6, 0.5) | -13.1 (-25.5, -0.8) | 0.037 |
| Social functioning | 7.7 (1.0, 14.3) | -10.9 (-18.1, -3.8) | -18.6 (-28.4, -8.8) | <.001 |
| Appetite | -1.8 (-8.0, 4.4) | 2.5 (-4.2, 9.1) | 4.3 (-4.8, 13.4) | 0.35 |
| Constipation | 9.3 (3.1, 15.5) | 7.7 (1.0, 14.4) | -1.6 (-10.7, 7.6) | 0.74 |
| Diarrhoea | 2.7 (-2.3, 7.6) | 3.5 (-1.8, 8.9) | 0.9 (-6.4, 8.2) | 0.81 |
| Dyspnoea | 7.3 (0.6, 14.0) | 6.4 (-0.9, 13.7) | -0.9 (-10.8, 9.0) | 0.85 |
| Fatigue | 9.5 (3.6, 15.4) | 14.5 (8.1, 20.9) | 5.0 (-3.6, 13.7) | 0.26 |
| Financial difficulties | -4.6 (-10.7, 1.6) | -2.2 (-8.8, 4.5) | 2.4 (-6.6, 11.4) | 0.60 |
| Nausea | 1.9 (-1.8, 5.6) | 1.4 (-2.6, 5.3) | -0.6 (-6.0, 4.8) | 0.83 |
| Pain | 7.4 (1.5, 13.3) | 9.4 (3.0, 15.7) | 2.0 (-6.7, 10.6) | 0.66 |
| Insomnia | 9.8 (2.1, 17.6) | 14.3 (5.9, 22.6) | 4.4 (-7.0, 15.8) | 0.45 |
| QLQ-C30 Summary Score | -3.2 (-6.1, -0.2) | -7.1 (-10.2, -3.9) | -3.9 (-8.2, 0.5) | 0.081 |

**Table 2.1.7 Model-Estimated Difference Baseline-60m between Education Categories (95% CI) from the MMRM Analysis**

| **Variable** | **Change** **Low Mid Education** | **Change** **Higher education** | **Change Difference** | **p-value** |
| --- | --- | --- | --- | --- |
| Cognitive functioning | -6.3 (-11.4, -1.3) | -7.3 (-13.5, -1.1) | -1.0 (-9.0, 7.1) | 0.81 |
| Emotional functioning | 5.5 (0.8, 10.2) | 0.7 (-5.0, 6.5) | -4.8 (-12.2, 2.6) | 0.20 |
| Global Health Status | -2.2 (-7.4, 3.0) | -4.7 (-11.0, 1.7) | -2.5 (-10.7, 5.7) | 0.55 |
| Physical functioning | -4.4 (-8.1, -0.7) | -8.2 (-12.7, -3.7) | -3.9 (-9.7, 2.0) | 0.19 |
| Role functioning | 0.8 (-7.2, 8.8) | -4.5 (-14.2, 5.3) | -5.3 (-17.9, 7.4) | 0.41 |
| Social functioning | -1.9 (-8.3, 4.4) | 1.7 (-6.1, 9.5) | 3.6 (-6.4, 13.7) | 0.48 |
| Appetite | -1.1 (-6.9, 4.8) | 0.8 (-6.3, 8.0) | 1.9 (-7.4, 11.2) | 0.69 |
| Constipation | 5.2 (-0.7, 11.1) | 11.8 (4.6, 19.0) | 6.6 (-2.7, 15.9) | 0.16 |
| Diarrhoea | 4.8 (0.1, 9.5) | -0.2 (-5.9, 5.6) | -5.0 (-12.4, 2.4) | 0.19 |
| Dyspnoea | 2.8 (-3.6, 9.2) | 12.5 (4.5, 20.4) | 9.7 (-0.5, 19.9) | 0.063 |
| Fatigue | 10.3 (4.6, 15.9) | 13.4 (6.5, 20.3) | 3.1 (-5.8, 12.1) | 0.49 |
| Financial difficulties | -8.2 (-14.0, -2.4) | 1.4 (-5.7, 8.6) | 9.6 (0.4, 18.8) | 0.041 |
| Nausea | 0.4 (-3.1, 3.8) | 3.3 (-0.9, 7.5) | 2.9 (-2.5, 8.4) | 0.29 |
| Pain | 5.2 (-0.4, 10.8) | 11.8 (5.0, 18.7) | 6.6 (-2.2, 15.5) | 0.14 |
| Insomnia | 11.8 (4.5, 19.2) | 11.8 (2.8, 20.8) | -0.1 (-11.7, 11.5) | 0.99 |
| QLQ-C30 Summary Score | -3.6 (-6.4, -0.8) | -6.3 (-9.7, -2.9) | -2.7 (-7.1, 1.7) | 0.22 |

**Table 2.2.1 BN20**

| **Variable** | **Baseline (n=126)** | **Mid (n=126)** | **End (n=126)** | **1m (n=126)** | **3m (n=126)** | **6m (n=126)** | **9m (n=126)** | **12m (n=126)** | **24m (n=126)** | **36m (n=126)** | **60m (n=126)** |
| --- | --- | --- | --- | --- | --- | --- | --- | --- | --- | --- | --- |
| **Future uncertainty** | 20.8 (22.1) n=120 | 20.1 (21.2) n=119 | 20.8 (21.5) n=117 | 20.7 (21.6) n=117 | 22.0 (22.9) n=116 | 18.8 (20.6) n=111 | 19.5 (20.1) n=111 | 16.0 (18.9) n=102 | 18.2 (20.7) n=105 | 19.7 (20.1) n=91 | 16.2 (18.8) n=77 |
| **Visual disorder** | 14.5 (21.5) n=120 | 15.4 (21.9) n=119 | 17.1 (22.1) n=117 | 16.7 (23.0) n=116 | 17.0 (24.7) n=116 | 15.2 (20.0) n=111 | 15.3 (20.0) n=111 | 17.3 (22.5) n=102 | 16.0 (21.8) n=105 | 16.0 (20.4) n=91 | 12.4 (18.4) n=77 |
| **Motor dysfunction** | 8.38 (13.63) n=120 | 9.71 (15.55) n=119 | 11.0 (17.2) n=117 | 13.1 (20.9) n=116 | 13.2 (18.3) n=116 | 12.4 (18.7) n=111 | 11.6 (18.4) n=111 | 11.2 (17.4) n=102 | 11.9 (17.7) n=105 | 11.5 (15.6) n=91 | 12.7 (14.0) n=77 |
| **Communication deficit** | 9.81 (16.44) n=120 | 12.0 (17.9) n=119 | 13.5 (18.6) n=117 | 12.8 (18.5) n=116 | 13.5 (17.0) n=116 | 13.3 (18.9) n=111 | 12.4 (16.2) n=111 | 12.3 (16.0) n=102 | 11.9 (15.0) n=105 | 13.3 (15.4) n=91 | 12.4 (16.4) n=77 |
| **Headaches** | 26.4 (27.3) n=120 | 31.7 (28.1) n=119 | 30.8 (30.7) n=117 | 31.6 (30.6) n=117 | 30.5 (31.6) n=116 | 27.3 (30.2) n=111 | 24.0 (26.7) n=111 | 25.8 (28.1) n=102 | 23.2 (29.3) n=105 | 20.9 (26.1) n=91 | 20.3 (27.1) n=77 |
| **Seizures.** | 1.11 (7.40) n=120 | 0.280 (3.056) n=119 | 0.862 (6.896) n=116 | 3.16 (14.57) n=116 | 1.45 (8.13) n=115 | 2.10 (12.93) n=111 | 1.80 (10.86) n=111 | 0.980 (5.660) n=102 | 1.90 (9.04) n=105 | 1.83 (9.11) n=91 | 0.866 (5.337) n=77 |
| **Drowsiness** | 30.8 (24.9) n=120 | 33.9 (26.4) n=119 | 42.5 (29.2) n=117 | 40.2 (30.6) n=116 | 41.1 (29.3) n=116 | 34.2 (28.6) n=111 | 33.9 (27.7) n=111 | 35.6 (29.8) n=102 | 36.5 (29.8) n=105 | 34.4 (29.6) n=91 | 37.7 (28.8) n=77 |
| **Itchy skin** | 5.83 (16.55) n=120 | 12.3 (22.1) n=119 | 12.3 (23.0) n=117 | 9.48 (19.56) n=116 | 9.48 (19.06) n=116 | 8.71 (17.82) n=111 | 8.11 (16.95) n=111 | 7.84 (17.03) n=102 | 10.8 (21.4) n=105 | 11.4 (20.6) n=91 | 11.7 (19.3) n=77 |
| **Hair loss** | 2.78 (10.21) n=120 | 12.3 (26.7) n=119 | 18.8 (29.8) n=117 | 18.4 (26.9) n=116 | 17.2 (28.0) n=116 | 13.6 (24.9) n=110 | 7.81 (19.57) n=111 | 7.19 (17.32) n=102 | 9.52 (22.51) n=105 | 9.16 (23.34) n=91 | 12.1 (21.6) n=77 |
| **Weakness of legs** | 5.28 (14.97) n=120 | 8.68 (19.63) n=119 | 9.97 (22.43) n=117 | 12.4 (24.3) n=116 | 11.3 (20.7) n=115 | 9.61 (20.29) n=111 | 7.51 (18.36) n=111 | 8.17 (18.43) n=102 | 12.1 (22.7) n=105 | 9.52 (20.05) n=91 | 8.66 (18.26) n=77 |
| **Bladder control** | 6.11 (15.57) n=120 | 5.04 (15.43) n=119 | 7.12 (17.96) n=117 | 8.33 (20.08) n=116 | 9.48 (20.99) n=116 | 9.61 (20.79) n=111 | 11.4 (23.6) n=111 | 10.2 (20.4) n=101 | 11.7 (22.2) n=105 | 10.3 (23.1) n=91 | 12.6 (23.6) n=77 |
| For continuous variables Mean (SD) / n= is presented.   Effect size is estimated using the Cohen method (difference in mean/SD at first timepoint). | | | | | | | | | | | |

**Table 2.2.2 Change in BN20**

| **Variable** | **Change from Baseline to Mid** | **Change from Baseline to End** | **Change from Baseline to 1m** | **Change from Baseline to 3m** | **Change from Baseline to 6m** | **Change from Baseline to 9m** | **Change from Baseline to 12m** | **Change from Baseline to 24m** | **Change from Baseline to 36m** | **Change from Baseline to 60m** |
| --- | --- | --- | --- | --- | --- | --- | --- | --- | --- | --- |
| **Future uncertainty** | -1.48 (-4.76; 1.79) n=116 p=0.37 ES=-0.07 | -0.145 (-3.472; 3.205) n=115 p=0.93 ES=-0.01 | -0.219 (-3.774; 3.302) n=114 p=0.90 ES=-0.01 | 1.23 (-2.31; 4.69) n=115 p=0.49 ES=0.06 | -1.52 (-5.36; 2.29) n=110 p=0.43 ES=-0.07 | -0.076 (-4.096; 3.993) n=109 p=0.97 ES=-0.00 | -3.03 (-7.55; 1.50) n=100 p=0.19 ES=-0.15 | -1.57 (-5.33; 2.24) n=101 p=0.42 ES=-0.07 | 0.741 (-4.000; 5.492) n=90 p=0.76 ES=0.04 | -2.19 (-7.64; 3.42) n=76 p=0.43 ES=-0.11 |
| **Visual disorder** | 0.479 (-1.923; 2.889) n=116 p=0.70 ES=0.02 | 2.61 (-0.19; 5.43) n=115 p=0.063 ES=0.12 | 2.44 (-0.67; 5.56) n=114 p=0.13 ES=0.11 | 2.71 (-0.61; 6.03) n=115 p=0.11 ES=0.12 | 0.808 (-2.330; 3.909) n=110 p=0.61 ES=0.04 | 1.43 (-2.35; 5.16) n=109 p=0.46 ES=0.07 | 3.33 (-0.93; 7.63) n=100 p=0.13 ES=0.16 | 2.09 (-2.27; 6.54) n=101 p=0.35 ES=0.10 | 1.73 (-3.14; 6.62) n=90 p=0.49 ES=0.08 | -1.90 (-6.84; 3.06) n=76 p=0.45 ES=-0.09 |
| **Motor dysfunction** | 0.910 (-1.462; 3.268) n=116 p=0.46 ES=0.07 | 2.66 (0.10; 5.18) n=115 p=0.042 ES=0.19 | 5.02 (2.13; 7.91) n=114 p=0.0008 ES=0.37 | 5.17 (2.49; 7.80) n=115 p=0.0002 ES=0.39 | 4.14 (1.64; 6.63) n=110 p=0.0015 ES=0.30 | 3.72 (0.69; 6.80) n=109 p=0.019 ES=0.27 | 3.72 (0.34; 7.07) n=100 p=0.031 ES=0.28 | 3.36 (0.19; 6.56) n=101 p=0.041 ES=0.24 | 4.88 (1.59; 8.15) n=90 p=0.0042 ES=0.45 | 6.36 (3.23; 9.49) n=76 p=0.0001 ES=0.59 |
| **Communication deficit** | 2.11 (-0.61; 4.82) n=116 p=0.12 ES=0.13 | 4.15 (1.23; 7.09) n=115 p=0.0059 ES=0.26 | 4.19 (1.09; 7.28) n=114 p=0.0097 ES=0.28 | 4.15 (1.41; 6.86) n=115 p=0.0031 ES=0.26 | 3.43 (0.20; 6.71) n=110 p=0.040 ES=0.21 | 2.91 (0.00; 5.77) n=109 p=0.046 ES=0.18 | 3.11 (0.44; 5.91) n=100 p=0.028 ES=0.21 | 2.86 (0.19; 5.66) n=101 p=0.042 ES=0.19 | 5.06 (1.78; 8.33) n=90 p=0.0027 ES=0.39 | 4.39 (1.08; 7.75) n=76 p=0.010 ES=0.32 |
| **Headaches** | 4.89 (0.68; 8.93) n=116 p=0.019 ES=0.18 | 4.06 (-0.62; 8.81) n=115 p=0.094 ES=0.15 | 4.68 (0.00; 9.36) n=114 p=0.048 ES=0.17 | 3.48 (-1.17; 8.16) n=115 p=0.14 ES=0.13 | 1.52 (-3.27; 6.29) n=110 p=0.53 ES=0.06 | -1.83 (-6.29; 2.56) n=109 p=0.42 ES=-0.07 | 0.333 (-4.575; 5.333) n=100 p=0.89 ES=0.01 | -3.63 (-8.16; 0.76) n=101 p=0.12 ES=-0.13 | -5.56 (-9.91; -1.33) n=90 p=0.013 ES=-0.21 | -5.26 (-11.11; 0.81) n=76 p=0.077 ES=-0.19 |
| **Seizures.** | -1.15 (-2.42; -0.00) n=116 p=0.10 ES=-0.15 | -0.292 (-1.887; 1.282) n=114 p=0.74 ES=-0.04 | 2.05 (-1.11; 5.13) n=114 p=0.19 ES=0.27 | 0.292 (-1.212; 1.754) n=114 p=0.71 ES=0.04 | 0.909 (-1.923; 3.763) n=110 p=0.53 ES=0.12 | 0.612 (-1.852; 3.086) n=109 p=0.64 ES=0.08 | -0.333 (-2.041; 1.361) n=100 p=0.71 ES=-0.04 | 0.660 (-1.481; 2.899) n=101 p=0.57 ES=0.08 | 1.11 (-0.85; 3.25) n=90 p=0.32 ES=0.22 | -0.877 (-2.857; 0.980) n=76 p=0.42 ES=-0.09 |
| **Drowsiness** | 2.87 (-1.19; 6.92) n=116 p=0.17 ES=0.12 | 11.9 (7.0; 16.9) n=115 p=<.0001 ES=0.47 | 10.2 (5.5; 14.9) n=114 p=<.0001 ES=0.41 | 10.7 (5.6; 15.8) n=115 p=<.0001 ES=0.43 | 3.94 (-0.67; 8.64) n=110 p=0.10 ES=0.16 | 3.67 (0.00; 7.64) n=109 p=0.070 ES=0.15 | 5.33 (0.63; 10.32) n=100 p=0.035 ES=0.22 | 6.27 (0.93; 11.52) n=101 p=0.020 ES=0.25 | 5.19 (-0.78; 11.11) n=90 p=0.090 ES=0.21 | 9.65 (2.94; 16.24) n=76 p=0.0052 ES=0.42 |
| **Itchy skin** | 6.61 (3.14; 10.17) n=116 p=0.0003 ES=0.39 | 6.09 (1.75; 10.42) n=115 p=0.0066 ES=0.36 | 4.09 (0.00; 8.19) n=114 p=0.052 ES=0.29 | 3.48 (-0.64; 7.65) n=115 p=0.10 ES=0.21 | 3.03 (-1.26; 7.32) n=110 p=0.17 ES=0.19 | 2.14 (-1.89; 6.06) n=109 p=0.29 ES=0.13 | 1.67 (-2.96; 6.35) n=100 p=0.48 ES=0.10 | 4.29 (0.00; 8.67) n=101 p=0.052 ES=0.24 | 5.93 (1.31; 10.67) n=90 p=0.015 ES=0.42 | 7.46 (2.27; 12.64) n=76 p=0.0057 ES=0.59 |
| **Hair loss** | 8.91 (4.40; 13.33) n=116 p=0.0002 ES=0.86 | 15.7 (10.4; 20.8) n=115 p=<.0001 ES=1.50 | 15.2 (10.5; 19.9) n=114 p=<.0001 ES=1.45 | 14.8 (10.0; 19.6) n=115 p=<.0001 ES=1.48 | 10.7 (5.9; 15.6) n=109 p=<.0001 ES=1.00 | 4.89 (1.19; 8.70) n=109 p=0.013 ES=0.46 | 4.33 (0.67; 8.05) n=100 p=0.023 ES=0.41 | 6.60 (2.08; 11.11) n=101 p=0.0050 ES=0.60 | 6.30 (1.33; 11.59) n=90 p=0.019 ES=0.61 | 11.0 (5.7; 16.2) n=76 p=0.0001 ES=1.68 |
| **Weakness of legs** | 2.87 (0.00; 5.82) n=116 p=0.058 ES=0.19 | 4.35 (0.00; 8.62) n=115 p=0.046 ES=0.29 | 7.31 (2.56; 12.02) n=114 p=0.0026 ES=0.52 | 6.43 (2.78; 10.12) n=114 p=0.0007 ES=0.45 | 4.55 (1.19; 8.02) n=110 p=0.011 ES=0.32 | 2.75 (-1.11; 6.48) n=109 p=0.15 ES=0.19 | 3.67 (0.00; 7.64) n=100 p=0.070 ES=0.27 | 6.93 (3.03; 10.85) n=101 p=0.0006 ES=0.48 | 6.67 (2.22; 11.11) n=90 p=0.0039 ES=0.70 | 5.26 (0.81; 10.00) n=76 p=0.027 ES=0.45 |
| **Bladder control** | -0.862 (-3.333; 1.695) n=116 p=0.49 ES=-0.06 | 1.45 (-1.56; 4.40) n=115 p=0.34 ES=0.10 | 3.22 (-0.55; 6.77) n=114 p=0.078 ES=0.24 | 4.06 (0.61; 7.53) n=115 p=0.022 ES=0.28 | 3.64 (0.00; 7.22) n=110 p=0.051 ES=0.24 | 6.12 (2.22; 10.06) n=109 p=0.0028 ES=0.44 | 5.05 (1.33; 8.89) n=99 p=0.011 ES=0.36 | 6.93 (2.67; 11.11) n=101 p=0.0018 ES=0.50 | 5.93 (0.76; 11.11) n=90 p=0.026 ES=0.48 | 7.46 (1.85; 12.96) n=76 p=0.0086 ES=0.56 |
| For continuous variables Mean (SD) is presented. For change in continuous variables Mean (95% CI for Mean using the inversion of Fisher´s non-parametric permutation test) / n= is presented.  For comparison over time, the t-test was used for continuous variables. Effect size is estimated using the Cohen method (difference in mean/SD at first timepoint). | | | | | | | | | | |

**Table 2.2.3 Model-Estimated Means (95% CI) for BN20 from the MMRM Analysis**

|  | **Visit** | | | | | | | | | | |
| --- | --- | --- | --- | --- | --- | --- | --- | --- | --- | --- | --- |
|  | **Baseline** | **Mid** | **End** | **1m** | **3m** | **6m** | **9m** | **12m** | **24m** | **36m** | **60m** |
| Bladder control | 5.7 (2.5, 8.9) | 4.7 (1.5, 8.0) | 7.1 (3.8, 10.4) | 8.8 (5.5, 12.0) | 9.6 (6.4, 12.9) | 9.3 (6.0, 12.6) | 11.5 (8.2, 14.9) | 10.8 (7.4, 14.3) | 12.6 (9.1, 16.0) | 12.6 (9.0, 16.2) | 13.2 (9.4, 17.0) |
| Communication deficit | 9.5 (7.1, 11.9) | 11.4 (9.0, 13.9) | 13.4 (11.0, 15.9) | 13.2 (10.7, 15.6) | 13.2 (10.8, 15.7) | 13.1 (10.6, 15.6) | 13.2 (10.7, 15.7) | 13.1 (10.5, 15.6) | 12.4 (9.9, 15.0) | 14.1 (11.4, 16.8) | 14.4 (11.7, 17.2) |
| Drowsiness | 30.7 (26.6, 34.7) | 33.8 (29.7, 37.9) | 42.9 (38.8, 47.0) | 40.1 (36.0, 44.2) | 41.3 (37.1, 45.4) | 34.7 (30.5, 38.9) | 34.7 (30.5, 38.9) | 35.7 (31.3, 40.0) | 37.0 (32.6, 41.3) | 34.9 (30.3, 39.4) | 40.0 (35.2, 44.8) |
| Future uncertainty | 20.4 (17.4, 23.4) | 19.1 (16.1, 22.2) | 20.5 (17.4, 23.5) | 20.1 (17.0, 23.1) | 21.5 (18.4, 24.5) | 18.7 (15.6, 21.8) | 20.1 (17.0, 23.2) | 16.7 (13.5, 19.9) | 18.5 (15.3, 21.7) | 20.8 (17.4, 24.1) | 18.9 (15.5, 22.4) |
| Hair loss | 2.8 (-1.2, 6.8) | 11.9 (7.8, 15.9) | 18.6 (14.6, 22.6) | 17.9 (13.9, 22.0) | 17.4 (13.4, 21.4) | 13.6 (9.4, 17.7) | 8.3 (4.1, 12.4) | 8.1 (3.9, 12.4) | 9.8 (5.5, 14.1) | 9.7 (5.2, 14.1) | 14.2 (9.6, 18.8) |
| Headaches | 26.3 (22.5, 30.1) | 31.3 (27.5, 35.2) | 30.6 (26.8, 34.5) | 31.1 (27.2, 35.0) | 29.8 (26.0, 33.7) | 27.8 (23.9, 31.7) | 24.3 (20.4, 28.3) | 27.0 (22.9, 31.1) | 22.4 (18.4, 26.5) | 20.7 (16.5, 25.0) | 21.3 (16.8, 25.7) |
| Itchy skin | 5.8 (2.5, 9.1) | 12.6 (9.2, 15.9) | 12.0 (8.7, 15.4) | 9.5 (6.1, 12.9) | 9.3 (5.9, 12.7) | 9.0 (5.6, 12.4) | 8.6 (5.2, 12.1) | 7.8 (4.2, 11.4) | 10.8 (7.3, 14.4) | 12.1 (8.4, 15.8) | 13.4 (9.5, 17.3) |
| Motor dysfunction | 8.1 (5.7, 10.5) | 9.0 (6.5, 11.5) | 10.7 (8.2, 13.2) | 12.9 (10.5, 15.4) | 13.0 (10.6, 15.5) | 12.4 (9.9, 14.9) | 12.2 (9.6, 14.7) | 11.9 (9.3, 14.5) | 11.4 (8.8, 14.0) | 13.0 (10.3, 15.7) | 14.8 (12.0, 17.6) |
| Seizures | 1.1 (-0.5, 2.7) | 0.0 (-1.6, 1.7) | 0.9 (-0.7, 2.6) | 3.3 (1.6, 4.9) | 1.4 (-0.2, 3.1) | 2.1 (0.4, 3.8) | 1.8 (0.1, 3.5) | 1.2 (-0.6, 2.9) | 1.9 (0.2, 3.7) | 1.8 (-0.0, 3.7) | 0.7 (-1.3, 2.7) |
| Visual disorder | 14.4 (11.6, 17.3) | 15.0 (12.1, 18.0) | 17.2 (14.3, 20.2) | 16.7 (13.8, 19.6) | 17.0 (14.1, 19.9) | 15.3 (12.3, 18.2) | 15.9 (12.9, 18.9) | 18.0 (14.9, 21.1) | 16.6 (13.5, 19.7) | 16.7 (13.5, 19.9) | 13.6 (10.3, 17.0) |
| Weakness of legs | 5.0 (1.7, 8.3) | 7.9 (4.5, 11.2) | 9.6 (6.3, 13.0) | 11.9 (8.6, 15.3) | 11.1 (7.7, 14.5) | 9.9 (6.5, 13.3) | 7.8 (4.3, 11.2) | 8.6 (5.0, 12.1) | 11.6 (8.1, 15.2) | 11.8 (8.1, 15.5) | 11.2 (7.3, 15.2) |

**Table 2.2.4 Model-Estimated Difference from Baseline (95% CI) for BN20 Subscales from the MMRM Analysis**

|  | **Visit** | | | | | | | | | |
| --- | --- | --- | --- | --- | --- | --- | --- | --- | --- | --- |
|  | **Mid** | **End** | **1m** | **3m** | **6m** | **9m** | **12m** | **24m** | **36m** | **60m** |
| Bladder control | -0.9 (-5.5, 3.6) p 0.69 | 1.4 (-3.1, 6.0) p 0.54 | 3.1 (-1.5, 7.7) p 0.18 | 4.0 (-0.6, 8.5) p 0.086 | 3.6 (-1.0, 8.2) p 0.12 | 5.9 (1.3, 10.5) p 0.012 | 5.2 (0.6, 9.8) p 0.028 | 6.9 (2.4, 11.4) p 0.003 | 6.9 (2.6, 11.2) p 0.002 | 7.5 (3.7, 11.4) p <.001 |
| Communication deficit | 2.0 (-1.5, 5.4) p 0.26 | 4.0 (0.6, 7.4) p 0.023 | 3.7 (0.3, 7.1) p 0.032 | 3.8 (0.4, 7.1) p 0.029 | 3.7 (0.3, 7.0) p 0.034 | 3.7 (0.4, 7.0) p 0.030 | 3.6 (0.3, 6.9) p 0.031 | 3.0 (-0.2, 6.1) p 0.062 | 4.6 (1.7, 7.6) p 0.002 | 5.0 (2.5, 7.5) p <.001 |
| Drowsiness | 3.2 (-2.6, 8.9) p 0.28 | 12.3 (6.5, 18.0) p <.001 | 9.4 (3.7, 15.2) p 0.001 | 10.6 (4.9, 16.4) p <.001 | 4.1 (-1.7, 9.8) p 0.17 | 4.0 (-1.7, 9.8) p 0.17 | 5.0 (-0.8, 10.8) p 0.089 | 6.3 (0.7, 11.9) p 0.028 | 4.2 (-1.2, 9.6) p 0.12 | 9.3 (4.6, 14.1) p <.001 |
| Future uncertainty | -1.3 (-5.5, 3.0) p 0.56 | 0.1 (-4.2, 4.3) p 0.98 | -0.4 (-4.6, 3.9) p 0.87 | 1.0 (-3.2, 5.2) p 0.63 | -1.7 (-5.9, 2.5) p 0.42 | -0.3 (-4.5, 3.9) p 0.90 | -3.7 (-7.9, 0.4) p 0.077 | -1.9 (-5.9, 2.0) p 0.34 | 0.3 (-3.4, 4.1) p 0.86 | -1.5 (-4.7, 1.7) p 0.36 |
| Hair loss | 9.1 (3.4, 14.7) p 0.002 | 15.8 (10.1, 21.5) p <.001 | 15.1 (9.5, 20.8) p <.001 | 14.6 (9.0, 20.2) p <.001 | 10.8 (5.1, 16.4) p <.001 | 5.5 (-0.1, 11.0) p 0.054 | 5.3 (-0.2, 10.9) p 0.059 | 7.0 (1.7, 12.3) p 0.010 | 6.9 (1.9, 11.9) p 0.007 | 11.4 (7.1, 15.7) p <.001 |
| Headaches | 5.0 (-0.4, 10.4) p 0.069 | 4.3 (-1.1, 9.7) p 0.12 | 4.8 (-0.6, 10.2) p 0.083 | 3.5 (-1.9, 8.9) p 0.20 | 1.5 (-3.9, 6.9) p 0.59 | -2.0 (-7.4, 3.4) p 0.47 | 0.7 (-4.8, 6.1) p 0.81 | -3.9 (-9.1, 1.4) p 0.15 | -5.6 (-10.6, -0.5) p 0.030 | -5.0 (-9.5, -0.5) p 0.028 |
| Itchy skin | 6.8 (2.0, 11.5) p 0.005 | 6.2 (1.5, 11.0) p 0.010 | 3.7 (-1.0, 8.4) p 0.13 | 3.5 (-1.2, 8.2) p 0.15 | 3.2 (-1.5, 7.9) p 0.19 | 2.8 (-1.9, 7.5) p 0.24 | 2.0 (-2.7, 6.7) p 0.41 | 5.0 (0.5, 9.6) p 0.031 | 6.3 (1.9, 10.6) p 0.005 | 7.6 (3.7, 11.5) p <.001 |
| Motor dysfunction | 0.9 (-2.5, 4.4) p 0.60 | 2.6 (-0.9, 6.1) p 0.14 | 4.8 (1.4, 8.3) p 0.006 | 4.9 (1.5, 8.4) p 0.005 | 4.3 (0.9, 7.8) p 0.013 | 4.1 (0.7, 7.5) p 0.019 | 3.8 (0.4, 7.2) p 0.026 | 3.3 (0.1, 6.6) p 0.045 | 4.9 (1.8, 7.9) p 0.002 | 6.7 (4.1, 9.3) p <.001 |
| Seizures | -1.1 (-3.4, 1.2) p 0.34 | -0.2 (-2.5, 2.1) p 0.86 | 2.1 (-0.2, 4.5) p 0.071 | 0.3 (-2.0, 2.6) p 0.79 | 1.0 (-1.4, 3.3) p 0.42 | 0.7 (-1.7, 3.0) p 0.56 | 0.0 (-2.4, 2.4) p 0.97 | 0.8 (-1.6, 3.2) p 0.51 | 0.7 (-1.7, 3.1) p 0.57 | -0.5 (-2.8, 1.9) p 0.71 |
| Visual disorder | 0.6 (-3.5, 4.7) p 0.78 | 2.8 (-1.3, 6.9) p 0.18 | 2.3 (-1.8, 6.4) p 0.28 | 2.5 (-1.5, 6.6) p 0.22 | 0.8 (-3.3, 4.9) p 0.69 | 1.5 (-2.6, 5.6) p 0.47 | 3.6 (-0.5, 7.7) p 0.084 | 2.1 (-1.8, 6.1) p 0.29 | 2.3 (-1.5, 6.1) p 0.24 | -0.8 (-4.1, 2.5) p 0.63 |
| Weakness of legs | 2.9 (-1.8, 7.6) p 0.23 | 4.6 (-0.1, 9.3) p 0.053 | 6.9 (2.2, 11.6) p 0.004 | 6.1 (1.4, 10.8) p 0.011 | 4.9 (0.2, 9.7) p 0.041 | 2.8 (-2.0, 7.5) p 0.25 | 3.6 (-1.1, 8.3) p 0.14 | 6.6 (2.0, 11.2) p 0.005 | 6.8 (2.4, 11.3) p 0.003 | 6.3 (2.3, 10.2) p 0.002 |

**Table 2.2.5 Model-Estimated Difference Baseline-60m between Men and Women (95% CI) from the MMRM Analysis BN20**

| **Variable** | **Change Men** | **Change Women** | **Change Difference** | **p-value** |
| --- | --- | --- | --- | --- |
| Bladder control | 8.3 (0.6, 16.0) | 8.1 (3.0, 13.2) | -0.2 (-9.4, 9.1) | 0.97 |
| Communication deficit | 8.6 (3.4, 13.9) | 4.0 (0.5, 7.5) | -4.6 (-10.9, 1.7) | 0.15 |
| Drowsiness | 12.2 (2.8, 21.6) | 10.6 (4.3, 16.9) | -1.6 (-12.9, 9.8) | 0.79 |
| Future uncertainty | -4.6 (-11.0, 1.8) | 0.7 (-3.6, 5.0) | 5.3 (-2.4, 13.1) | 0.18 |
| Headaches | -0.6 (-9.5, 8.3) | -5.1 (-11.0, 0.9) | -4.4 (-15.2, 6.3) | 0.42 |
| Hair loss | 5.4 (-3.9, 14.7) | 15.5 (9.2, 21.7) | 10.1 (-1.1, 21.3) | 0.077 |
| Itchy skin | 7.8 (-0.5, 16.1) | 7.9 (2.3, 13.4) | 0.1 (-9.9, 10.0) | 0.99 |
| Motor dysfunction | 6.6 (1.1, 12.0) | 7.9 (4.2, 11.5) | 1.3 (-5.3, 7.9) | 0.70 |
| Seizures | -2.2 (-6.6, 2.2) | 0.9 (-2.0, 3.9) | 3.1 (-2.2, 8.5) | 0.25 |
| Visual disorder | -1.4 (-8.6, 5.8) | -0.6 (-5.4, 4.2) | 0.8 (-7.9, 9.5) | 0.85 |
| Weakness of legs | 1.1 (-6.6, 8.9) | 9.5 (4.3, 14.7) | 8.4 (-0.9, 17.8) | 0.078 |

**Table 2.2.6 Model-Estimated Difference Baseline-60m between Age Categories (95% CI) from the MMRM Analysis**

| **Variable** | **Change <60** | **Change >=60** | **Change Difference** | **p-value** |
| --- | --- | --- | --- | --- |
| Bladder control | 2.3 (-3.5, 8.2) | 14.5 (8.4, 20.7) | 12.2 (3.7, 20.7) | 0.005 |
| Communication deficit | 6.3 (2.3, 10.3) | 4.2 (-0.1, 8.4) | -2.1 (-8.0, 3.7) | 0.47 |
| Drowsiness | 12.0 (4.8, 19.2) | 10.0 (2.4, 17.7) | -1.9 (-12.5, 8.6) | 0.72 |
| Future uncertainty | -1.7 (-6.6, 3.2) | 0.0 (-5.2, 5.3) | 1.8 (-5.4, 8.9) | 0.63 |
| Headaches | -0.9 (-7.7, 5.9) | -6.7 (-13.9, 0.6) | -5.8 (-15.7, 4.2) | 0.26 |
| Hair loss | 10.2 (3.1, 17.3) | 14.3 (6.8, 21.9) | 4.2 (-6.2, 14.5) | 0.43 |
| Itchy skin | 7.6 (1.2, 13.9) | 7.8 (1.1, 14.6) | 0.3 (-9.0, 9.6) | 0.95 |
| Motor dysfunction | 5.6 (1.4, 9.7) | 9.4 (4.9, 13.8) | 3.8 (-2.3, 9.8) | 0.22 |
| Seizures | 0.0 (-3.4, 3.4) | -0.1 (-3.7, 3.6) | -0.1 (-5.0, 4.9) | 0.98 |
| Visual disorder | -2.4 (-7.9, 3.1) | 1.0 (-4.9, 6.8) | 3.4 (-4.6, 11.5) | 0.40 |
| Weakness of legs | 5.7 (-0.2, 11.7) | 8.2 (1.9, 14.5) | 2.4 (-6.2, 11.1) | 0.58 |

**Table 2.2.7 Model-Estimated Difference Baseline-60m between Education Categories (95% CI) from the MMRM Analysis**

| **Variable** | **Change Low Mid Education** | **Change Higher education** | **Change Difference** | **p-value** |
| --- | --- | --- | --- | --- |
| Bladder control | 11.7 (6.1, 17.3) | 3.1 (-3.7, 9.9) | -8.6 (-17.4, 0.2) | 0.054 |
| Communication deficit | 4.3 (0.5, 8.0) | 6.9 (2.3, 11.5) | 2.7 (-3.3, 8.6) | 0.38 |
| Drowsiness | 8.3 (1.5, 15.2) | 14.9 (6.6, 23.2) | 6.6 (-4.2, 17.3) | 0.23 |
| Future uncertainty | -3.6 (-8.2, 1.0) | 1.9 (-3.7, 7.5) | 5.5 (-1.8, 12.8) | 0.14 |
| Headaches | -4.4 (-10.8, 2.0) | -4.8 (-12.6, 3.0) | -0.3 (-10.4, 9.8) | 0.95 |
| Hair loss | 12.2 (5.5, 19.0) | 10.8 (2.7, 19.0) | -1.4 (-12.0, 9.2) | 0.80 |
| Itchy skin | 9.9 (3.9, 15.9) | 4.4 (-2.8, 11.7) | -5.5 (-14.9, 4.0) | 0.26 |
| Motor dysfunction | 8.7 (4.8, 12.7) | 5.3 (0.5, 10.1) | -3.4 (-9.6, 2.8) | 0.28 |
| Seizures | 0.2 (-3.0, 3.4) | -0.5 (-4.4, 3.5) | -0.7 (-5.8, 4.4) | 0.79 |
| Visual disorder | -1.8 (-7.0, 3.5) | 0.0 (-6.3, 6.4) | 1.8 (-6.4, 10.0) | 0.67 |
| Weakness of legs | 5.9 (0.3, 11.6) | 8.6 (1.7, 15.5) | 2.6 (-6.3, 11.6) | 0.56 |

**Table 3.1.1 Univariable Linear Regression of Physical Functioning 60m**

| **Variable** | **n missing** | **Value** | **Physical functioning 60m_n Mean (SD) Median n=** | **Beta (95% CI)** | **p-value** | **R-Square** |
| --- | --- | --- | --- | --- | --- | --- |
| **Age (Beta per 10 units)** | 0 | **22-<49** | 83.67 (21.02) 93.33 n=20 |  |  |  |
|  |  | **49-<64** | 86.42 (14.96) 93.33 n=27 |  |  |  |
|  |  | **64-80** | 76.22 (18.42) 80.00 n=30 | -2.28 (-5.56;1.01) | 0.17 | 0.02 |
| **Sex** | 0 | **Male** | 86.11 (14.44) 93.33 n=24 |  |  |  |
|  |  | **Female** | 79.75 (19.70) 86.67 n=53 | -6.36 (-15.31;2.58) | 0.16 | 0.03 |
| **Marital status** | 0 | **Married / Cohabiting (living with a partner)** | 80.79 (19.58) 86.67 n=59 |  |  |  |
|  |  | **Living alone (single household)** | 84.81 (13.68) 86.67 n=18 | 4.02 (-5.85;13.90) | 0.42 | 0.01 |
| **Employment status** | 0 | **Employed / Student** | 84.67 (17.37) 93.33 n=50 |  |  |  |
|  |  | **Retired / Disability pension / Unemployed / No occupation** | 76.30 (19.25) 80.00 n=27 | -8.37 (-16.95;0.21) | 0.056 | 0.05 |
| **Education level** | 1 | **Compulsory school** | 74.07 (13.52) 80.00 n=9 | Reference | 0.36*** |  |
|  |  | **Upper secondary / Vocational school** | 82.96 (16.67) 86.67 n=36 | 8.89 (-4.50;22.27) | 0.19 |  |
|  |  | **University / Higher education (College)** | 83.66 (20.43) 93.33 n=31 | 9.58 (-4.02;23.18) | 0.16 | 0.03 |
| All tests are performed with univariable linear regression. P-values, Beta and R-square are based on original values and not on stratified groups ***) p-value for the entire effect/factor/variable. | | | | | | |

**Table 3.1.2 Univariable Linear Regression of Change in Physical Functioning (BL–60m)**

| **Variable** | **n missing** | **Value** | **Change in Physical functioning (BL to 60m) Mean (SD) Median n=** | **Beta (95% CI)** | **p-value** | **R-Square** |
| --- | --- | --- | --- | --- | --- | --- |
| **Age (Beta per 10 units)** | 0 | **22-<49** | -6.33 (19.40) 0.00 n=20 |  |  |  |
|  |  | **49-<64** | -0.99 (11.80) 0.00 n=27 |  |  |  |
|  |  | **64-80** | -7.78 (12.27) -6.67 n=30 | -0.63 (-3.24;1.98) | 0.63 | 0.00 |
| **Sex** | 0 | **Male** | -4.72 (7.73) -3.33 n=24 |  |  |  |
|  |  | **Female** | -5.16 (16.67) 0.00 n=53 | -0.44 (-7.56;6.69) | 0.90 | 0.00 |
| **Marital status** | 0 | **Married / Cohabiting (living with a partner)** | -5.54 (14.86) 0.00 n=59 |  |  |  |
|  |  | **Living alone (single household)** | -3.33 (13.19) 0.00 n=18 | 2.20 (-5.57;9.98) | 0.57 | 0.00 |
| **Employment status** | 0 | **Employed / Student** | -3.47 (15.37) 0.00 n=50 |  |  |  |
|  |  | **Retired / Disability pension / Unemployed / No occupation** | -7.90 (12.27) -6.67 n=27 | -4.43 (-11.27;2.40) | 0.20 | 0.02 |
| **Education level** | 1 | **Compulsory school** | -6.67 (15.99) -6.67 n=9 | Reference | 0.57*** |  |
|  |  | **Upper secondary / Vocational school** | -2.78 (9.48) 0.00 n=36 | 3.89 (-6.53;14.31) | 0.46 |  |
|  |  | **University / Higher education (College)** | -6.02 (17.50) 0.00 n=31 | 0.65 (-9.94;11.24) | 0.90 | 0.02 |
| All tests are performed with univariable linear regression. P-values, Beta and R-square are based on original values and not on stratified groups ***) p-value for the entire effect/factor/variable. | | | | | | |

**Table 3.2.1 Univariable Linear Regression of Role Functioning 60m**

| **Variable** | **n missing** | **Value** | **Role functioning 60m_n Mean (SD) Median n=** | **Beta (95% CI)** | **p-value** | **R-Square** |
| --- | --- | --- | --- | --- | --- | --- |
| **Age (Beta per 10 units)** | 0 | **22-<49** | 73.33 (35.21) 91.67 n=20 |  |  |  |
|  |  | **49-<64** | 70.99 (32.22) 83.33 n=27 |  |  |  |
|  |  | **64-80** | 64.44 (35.49) 66.67 n=30 | -1.50 (-7.66;4.67) | 0.63 | 0.00 |
| **Sex** | 0 | **Male** | 72.92 (36.05) 100.00 n=24 |  |  |  |
|  |  | **Female** | 67.30 (33.33) 66.67 n=53 | -5.62 (-22.38;11.13) | 0.51 | 0.01 |
| **Marital status** | 0 | **Married / Cohabiting (living with a partner)** | 68.36 (35.79) 66.67 n=59 |  |  |  |
|  |  | **Living alone (single household)** | 71.30 (28.47) 83.33 n=18 | 2.93 (-15.45;21.31) | 0.75 | 0.00 |
| **Employment status** | 0 | **Employed / Student** | 73.00 (32.63) 83.33 n=50 |  |  |  |
|  |  | **Retired / Disability pension / Unemployed / No occupation** | 61.73 (36.05) 66.67 n=27 | -11.27 (-27.38;4.83) | 0.17 | 0.03 |
| **Education level** | 1 | **Compulsory school** | 55.56 (36.32) 66.67 n=9 | Reference | 0.41*** |  |
|  |  | **Upper secondary / Vocational school** | 72.22 (30.34) 83.33 n=36 | 16.67 (-8.43;41.76) | 0.19 |  |
|  |  | **University / Higher education (College)** | 70.97 (36.76) 100.00 n=31 | 15.41 (-10.08;40.91) | 0.23 | 0.02 |
| All tests are performed with univariable linear regression. P-values, Beta and R-square are based on original values and not on stratified groups ***) p-value for the entire effect/factor/variable. | | | | | | |

**Table 3.2.2** **Univariable Linear Regression of Change in Role Functioning (BL–60m)**

| **Variable** | **n missing** | **Value** | **Change in Role functioning (BL to 60m) Mean (SD) Median n=** | **Beta (95% CI)** | **p-value** | **R-Square** |  |  |
| --- | --- | --- | --- | --- | --- | --- | --- | --- |
| **Age (Beta per 10 units)** | 0 | **22-<49** | 14.17 (41.28) 16.67 n=20 |  |  |  |  |  |
|  |  | **49-<64** | 5.56 (37.55) 0.00 n=27 |  |  |  |  |  |
|  |  | **64-80** | -12.22 (31.54) -16.67 n=30 | -6.90 (-13.52;-0.29) | 0.041 | 0.05 |  |  |
| **Sex** | 0 | **Man** | 3.47 (42.56) 0.00 n=24 |  |  |  |  |  |
|  |  | **Male** | -0.31 (35.43) 0.00 n=53 | -3.79 (-22.29;14.72) | 0.68 | 0.00 |  |  |
| **Marital status** | 0 | **Female** | 1.41 (36.92) 0.00 n=59 |  |  |  |  |  |
|  |  | **Married / Cohabiting (living with a partner)** | -0.93 (40.61) -16.67 n=18 | -2.34 (-22.61;17.93) | 0.82 | 0.00 |  |  |
| **Employment status** | 0 | **Living alone (single household)** | 10.33 (39.54) 0.00 n=50 |  |  |  |  |  |
|  |  | **Employed / Student** | -16.67 (26.15) -16.67 n=27 | -27.00 (-43.88;-10.12) | 0.0021 | 0.12 |  |  |
| **Education level** | 1 | **Retired / Disability pension / Unemployed / No occupation** | -22.22 (37.27) -16.67 n=9 | Reference | 0.061*** |  |  |  |
|  |  | **Compulsory school** | 9.72 (33.42) 0.00 n=36 | 31.94 (4.89;59.00) | 0.021 |  |  |  |
|  |  | **Upper secondary / Vocational school** | -1.08 (39.43) 0.00 n=31 | 21.15 (-6.34;48.63) | 0.13 | 0.07 |  |  |
| All tests are performed with univariable linear regression. P-values, Beta and R-square are based on original values and not on stratified groups ***) p-value for the entire effect/factor/variable. | | | | | | |  | **University / Higher education (College)** |

**Table 3.3.1 Univariable Linear Regression of Emotional Functioning 60m**

| **Variable** | **n missing** | **Value** | **Emotional functioning 60m_n Mean (SD) Median n=** | **Beta (95% CI)** | **p-value** | **R-Square** |
| --- | --- | --- | --- | --- | --- | --- |
| **Age (Beta per 10 units)** | 0 | **22-<49** | 72.50 (18.75) 75.00 n=20 |  |  |  |
|  |  | **49-<64** | 86.11 (16.01) 91.67 n=27 |  |  |  |
|  |  | **64-80** | 82.22 (18.92) 83.33 n=30 | 3.03 (-0.24;6.30) | 0.069 | 0.04 |
| **Sex** | 0 | **Male** | 79.51 (19.50) 83.33 n=24 |  |  |  |
|  |  | **Female** | 81.76 (18.13) 83.33 n=53 | 2.25 (-6.85;11.34) | 0.62 | 0.00 |
| **Marital status** | 0 | **Married / Cohabiting (living with a partner)** | 80.79 (19.47) 83.33 n=59 |  |  |  |
|  |  | **Living alone (single household)** | 81.94 (15.19) 83.33 n=18 | 1.15 (-8.82;11.12) | 0.82 | 0.00 |
| **Employment status** | 0 | **Employed / Student** | 80.33 (18.73) 83.33 n=50 |  |  |  |
|  |  | **Retired / Disability pension / Unemployed / No occupation** | 82.41 (18.25) 83.33 n=27 | 2.07 (-6.76;10.91) | 0.64 | 0.00 |
| **Education level** | 1 | **Compulsory school** | 72.22 (16.67) 75.00 n=9 | Reference | 0.22*** |  |
|  |  | **Upper secondary / Vocational school** | 82.18 (18.38) 83.33 n=36 | 9.95 (-3.13;23.04) | 0.13 |  |
|  |  | **University / Higher education (College)** | 83.87 (16.93) 91.67 n=31 | 11.65 (-1.64;24.94) | 0.085 | 0.04 |
| All tests are performed with univariable linear regression. P-values, Beta and R-square are based on original values and not on stratified groups ***) p-value for the entire effect/factor/variable. | | | | | | |

**Table 3.3.2 Univariable Linear Regression of Change in Emotional Functioning (BL–60m)**

| **Variable** | **n missing** | **Value** | **Change in Emotional functioning (BL to 60m) Mean (SD) Median n=** | **Beta (95% CI)** | **p-value** | **R-Square** |
| --- | --- | --- | --- | --- | --- | --- |
| **Age (Beta per 10 units)** | 0 | **22-<49** | 2.08 (27.56) 0.00 n=20 |  |  |  |
|  |  | **49-<64** | 9.26 (18.97) 8.33 n=27 |  |  |  |
|  |  | **64-80** | 3.06 (19.14) 4.17 n=30 | -0.29 (-4.19;3.60) | 0.88 | 0.00 |
| **Sex** | 0 | **Male** | 5.21 (15.70) 4.17 n=24 |  |  |  |
|  |  | **Female** | 4.87 (23.82) 8.33 n=53 | -0.33 (-10.95;10.28) | 0.95 | 0.00 |
| **Marital status** | 0 | **Married / Cohabiting (living with a partner)** | 4.80 (18.71) 8.33 n=59 |  |  |  |
|  |  | **Living alone (single household)** | 5.56 (29.57) 4.17 n=18 | 0.75 (-10.86;12.37) | 0.90 | 0.00 |
| **Employment status** | 0 | **Employed / Student** | 5.33 (24.03) 8.33 n=50 |  |  |  |
|  |  | **Retired / Disability pension / Unemployed / No occupation** | 4.32 (16.24) 0.00 n=27 | -1.01 (-11.31;9.29) | 0.85 | 0.00 |
| **Education level** | 1 | **Compulsory school** | -0.93 (15.28) 0.00 n=9 | Reference | 0.28*** |  |
|  |  | **Upper secondary / Vocational school** | 9.49 (24.08) 8.33 n=36 | 10.42 (-4.89;25.73) | 0.18 |  |
|  |  | **University / Higher education (College)** | 3.23 (17.17) 0.00 n=31 | 4.15 (-11.40;19.71) | 0.60 | 0.03 |
| All tests are performed with univariable linear regression. P-values, Beta and R-square are based on original values and not on stratified groups ***) p-value for the entire effect/factor/variable. | | | | | | |

**Table 3.4.1 Univariable Linear Regression of Cognitive Functioning 60m**

| **Variable** | **n missing** | **Value** | **Cognitive functioning 60m_n Mean (SD) Median n=** | **Beta (95% CI)** | **p-value** | **R-Square** |
| --- | --- | --- | --- | --- | --- | --- |
| **Age (Beta per 10 units)** | 0 | **22-<49** | 66.67 (25.93) 75.00 n=20 |  |  |  |
|  |  | **49-<64** | 79.63 (19.79) 83.33 n=27 |  |  |  |
|  |  | **64-80** | 78.89 (18.54) 83.33 n=30 | 3.76 (-0.04;7.57) | 0.053 | 0.05 |
| **Sex** | 0 | **Male** | 72.92 (18.92) 83.33 n=24 |  |  |  |
|  |  | **Female** | 77.36 (22.67) 83.33 n=53 | 4.44 (-6.14;15.02) | 0.41 | 0.01 |
| **Marital status** | 0 | **Married / Cohabiting (living with a partner)** | 75.71 (21.29) 83.33 n=59 |  |  |  |
|  |  | **Living alone (single household)** | 76.85 (22.97) 83.33 n=18 | 1.15 (-10.48;12.78) | 0.84 | 0.00 |
| **Employment status** | 0 | **Employed / Student** | 74.33 (22.13) 83.33 n=50 |  |  |  |
|  |  | **Retired / Disability pension / Unemployed / No occupation** | 79.01 (20.46) 83.33 n=27 | 4.68 (-5.58;14.94) | 0.37 | 0.01 |
| **Education level** | 1 | **Compulsory school** | 68.52 (19.44) 83.33 n=9 | Reference | 0.51*** |  |
|  |  | **Upper secondary / Vocational school** | 77.78 (18.69) 83.33 n=36 | 9.26 (-6.76;25.28) | 0.25 |  |
|  |  | **University / Higher education (College)** | 76.88 (24.97) 83.33 n=31 | 8.36 (-7.91;24.64) | 0.31 | 0.02 |
| All tests are performed with univariable linear regression. P-values, Beta and R-square are based on original values and not on stratified groups ***) p-value for the entire effect/factor/variable. | | | | | | |

**Table 3.4.2 Univariable Linear Regression of Change in Cognitive Functioning (BL–60m)**

| **Variable** | **n missing** | **Value** | **Change in Cognitive functioning (BL to 60m) Mean (SD) Median n=** | **Beta (95% CI)** | **p-value** | **R-Square** |
| --- | --- | --- | --- | --- | --- | --- |
| **Age (Beta per 10 units)** | 0 | **22-<49** | -7.50 (28.85) -8.33 n=20 |  |  |  |
|  |  | **49-<64** | -2.47 (21.53) 0.00 n=27 |  |  |  |
|  |  | **64-80** | -6.67 (16.14) 0.00 n=30 | -0.28 (-4.21;3.66) | 0.89 | 0.00 |
| **Sex** | 0 | **Male** | -7.64 (20.84) 0.00 n=24 |  |  |  |
|  |  | **Female** | -4.40 (22.21) 0.00 n=53 | 3.24 (-7.45;13.92) | 0.55 | 0.00 |
| **Marital status** | 0 | **Married / Cohabiting (living with a partner)** | -7.34 (22.16) 0.00 n=59 |  |  |  |
|  |  | **Living alone (single household)** | 0.93 (19.36) 0.00 n=18 | 8.27 (-3.29;19.83) | 0.16 | 0.03 |
| **Employment status** | 0 | **Employed / Student** | -4.33 (24.47) 0.00 n=50 |  |  |  |
|  |  | **Retired / Disability pension / Unemployed / No occupation** | -7.41 (15.56) 0.00 n=27 | -3.07 (-13.44;7.30) | 0.56 | 0.00 |
| **Education level** | 1 | **Compulsory school** | -14.81 (17.57) -16.67 n=9 | Reference | 0.36*** |  |
|  |  | **Upper secondary / Vocational school** | -3.24 (20.24) 0.00 n=36 | 11.57 (-4.62;27.77) | 0.16 |  |
|  |  | **University / Higher education (College)** | -4.84 (24.42) 0.00 n=31 | 9.98 (-6.48;26.43) | 0.23 | 0.03 |
| All tests are performed with univariable linear regression. P-values, Beta and R-square are based on original values and not on stratified groups ***) p-value for the entire effect/factor/variable. | | | | | | |

**Table 3.5.1 Univariable Linear Regression of** **Social Functioning 60m**

| **Variable** | **n missing** | **Value** | **Social functioning 60m_n Mean (SD) Median n=** | **Beta (95% CI)** | **p-value** | **R-Square** |
| --- | --- | --- | --- | --- | --- | --- |
| **Age (Beta per 10 units)** | 0 | **22-<49** | 76.67 (25.59) 83.33 n=20 |  |  |  |
|  |  | **49-<64** | 85.80 (21.53) 100.00 n=27 |  |  |  |
|  |  | **64-80** | 76.67 (29.56) 83.33 n=30 | -1.61 (-6.30;3.08) | 0.50 | 0.01 |
| **Sex** | 0 | **Male** | 75.69 (30.29) 83.33 n=24 |  |  |  |
|  |  | **Female** | 81.76 (23.86) 83.33 n=53 | 6.07 (-6.68;18.81) | 0.35 | 0.01 |
| **Marital status** | 0 | **Married / Cohabiting (living with a partner)** | 77.40 (27.29) 83.33 n=59 |  |  |  |
|  |  | **Living alone (single household)** | 87.96 (19.64) 100.00 n=18 | 10.56 (-3.25;24.38) | 0.13 | 0.03 |
| **Employment status** | 0 | **Employed / Student** | 83.00 (22.46) 91.67 n=50 |  |  |  |
|  |  | **Retired / Disability pension / Unemployed / No occupation** | 74.07 (31.12) 83.33 n=27 | -8.93 (-21.20;3.35) | 0.15 | 0.03 |
| **Education level** | 1 | **Compulsory school** | 59.26 (34.47) 66.67 n=9 | Reference | 0.022*** |  |
|  |  | **Upper secondary / Vocational school** | 83.80 (20.11) 83.33 n=36 | 24.54 (6.60;42.47) | 0.0080 |  |
|  |  | **University / Higher education (College)** | 83.33 (25.09) 100.00 n=31 | 24.07 (5.85;42.29) | 0.010 | 0.10 |
| All tests are performed with univariable linear regression. P-values, Beta and R-square are based on original values and not on stratified groups ***) p-value for the entire effect/factor/variable. | | | | | | |

**Table 3.5.2 Univariable Linear Regression of** **Change in** **Social Functioning (BL–60m)**

| **Variable** | **n missing** | **Value** | **Change in Social functioning (BL to 60m) Mean (SD) Median n=** | **Beta (95% CI)** | **p-value** | **R-Square** |
| --- | --- | --- | --- | --- | --- | --- |
| **Age (Beta per 10 units)** | 0 | **22-<49** | 10.00 (34.37) 16.67 n=20 |  |  |  |
|  |  | **49-<64** | 3.09 (23.59) 0.00 n=27 |  |  |  |
|  |  | **64-80** | -10.56 (32.01) 0.00 n=30 | -7.73 (-13.03;-2.43) | 0.0048 | 0.10 |
| **Sex** | 0 | **Male** | -1.39 (36.42) 0.00 n=24 |  |  |  |
|  |  | **Female** | -0.00 (28.31) 0.00 n=53 | 1.39 (-13.82;16.59) | 0.86 | 0.00 |
| **Marital status** | 0 | **Married / Cohabiting (living with a partner)** | -1.98 (33.63) 0.00 n=59 |  |  |  |
|  |  | **Living alone (single household)** | 4.63 (18.79) 0.00 n=18 | 6.61 (-9.97;23.18) | 0.43 | 0.01 |
| **Employment status** | 0 | **Employed / Student** | 5.00 (28.62) 0.00 n=50 |  |  |  |
|  |  | **Retired / Disability pension / Unemployed / No occupation** | -10.49 (32.73) 0.00 n=27 | -15.49 (-29.82;-1.17) | 0.034 | 0.06 |
| **Education level** | 1 | **Compulsory school** | -25.93 (37.37) -16.67 n=9 | Reference | 0.021*** |  |
|  |  | **Upper secondary / Vocational school** | 3.70 (23.94) 0.00 n=36 | 29.63 (7.85;51.40) | 0.0083 |  |
|  |  | **University / Higher education (College)** | 3.76 (32.41) 0.00 n=31 | 29.69 (7.57;51.81) | 0.0092 | 0.10 |
| All tests are performed with univariable linear regression. P-values, Beta and R-square are based on original values and not on stratified groups ***) p-value for the entire effect/factor/variable. | | | | | | |

**Table 3.6.1 Univariable Linear Regression of** **Global Health Status 60m**

| **Variable** | **n missing** | **Value** | **Global health status 60m_n Mean (SD) Median n=** | **Beta (95% CI)** | **p-value** | **R-Square** |
| --- | --- | --- | --- | --- | --- | --- |
| **Age (Beta per 10 units)** | 0 | **22-<49** | 65.00 (22.72) 66.67 n=20 |  |  |  |
|  |  | **49-<64** | 65.12 (19.20) 66.67 n=27 |  |  |  |
|  |  | **64-80** | 67.78 (21.63) 70.83 n=30 | 0.81 (-2.96;4.59) | 0.67 | 0.00 |
| **Sex** | 0 | **Male** | 68.40 (21.28) 66.67 n=24 |  |  |  |
|  |  | **Female** | 65.09 (20.80) 66.67 n=53 | -3.31 (-13.58;6.96) | 0.52 | 0.01 |
| **Marital status** | 0 | **Married / Cohabiting (living with a partner)** | 67.94 (21.82) 66.67 n=59 |  |  |  |
|  |  | **Living alone (single household)** | 60.19 (16.56) 58.33 n=18 | -7.75 (-18.88;3.37) | 0.17 | 0.03 |
| **Employment status** | 0 | **Employed / Student** | 66.33 (20.62) 66.67 n=50 |  |  |  |
|  |  | **Retired / Disability pension / Unemployed / No occupation** | 65.74 (21.72) 66.67 n=27 | -0.59 (-10.59;9.40) | 0.91 | 0.00 |
| **Education level** | 1 | **Compulsory school** | 53.70 (19.59) 50.00 n=9 | Reference | 0.086*** |  |
|  |  | **Upper secondary / Vocational school** | 69.68 (17.50) 70.83 n=36 | 15.97 (1.74;30.21) | 0.028 |  |
|  |  | **University / Higher education (College)** | 67.74 (20.83) 66.67 n=31 | 14.04 (-0.42;28.50) | 0.057 | 0.07 |
| All tests are performed with univariable linear regression. P-values, Beta and R-square are based on original values and not on stratified groups ***) p-value for the entire effect/factor/variable. | | | | | | |

**Table 3.6.2 Univariable Linear Regression of Change in Global Health Status (BL–60m)**

| **Variable** | **n missing** | **Value** | **Change in Global Health Status (BL to 60m) Mean (SD) Median n=** | **Beta (95% CI)** | **p-value** | **R-Square** |
| --- | --- | --- | --- | --- | --- | --- |
| **Age (Beta per 10 units)** | 0 | **22-<49** | 2.08 (24.46) 0.00 n=20 |  |  |  |
|  |  | **49-<64** | -4.01 (15.04) 0.00 n=27 |  |  |  |
|  |  | **64-80** | -2.50 (21.46) -4.17 n=30 | -0.44 (-4.10;3.22) | 0.81 | 0.00 |
| **Sex** | 0 | **Male** | 3.12 (21.95) 0.00 n=24 |  |  |  |
|  |  | **Female** | -4.09 (19.17) 0.00 n=53 | -7.21 (-17.05;2.62) | 0.15 | 0.03 |
| **Marital status** | 0 | **Married / Cohabiting (living with a partner)** | -0.14 (21.47) 0.00 n=59 |  |  |  |
|  |  | **Living alone (single household)** | -7.41 (14.54) 0.00 n=18 | -7.27 (-18.05;3.52) | 0.18 | 0.02 |
| **Employment status** | 0 | **Employed / Student** | -2.33 (19.56) 0.00 n=50 |  |  |  |
|  |  | **Retired / Disability pension / Unemployed / No occupation** | -0.93 (21.72) 0.00 n=27 | 1.41 (-8.27;11.08) | 0.77 | 0.00 |
| **Education level** | 1 | **Compulsory school** | -9.26 (19.74) -8.33 n=9 | Reference | 0.36*** |  |
|  |  | **Upper secondary / Vocational school** | 1.16 (17.04) 0.00 n=36 | 10.42 (-4.11;24.94) | 0.16 |  |
|  |  | **University / Higher education (College)** | -1.61 (22.09) -8.33 n=31 | 7.65 (-7.11;22.40) | 0.31 | 0.03 |
| All tests are performed with univariable linear regression. P-values, Beta and R-square are based on original values and not on stratified groups ***) p-value for the entire effect/factor/variable. | | | | | | |

**Table 4.1.1 Univariable Linear Regression of Future uncertainty**

| **Variable** | **n missing** | **Value** | **Future uncertainty 60m_n Mean (SD) Median n=** | **Beta (95% CI)** | **p-value** | **R-Square** |
| --- | --- | --- | --- | --- | --- | --- |
| **Age (Beta per 10 units)** | 0 | **22-<49** | 21.67 (18.02) 16.67 n=20 |  |  |  |
|  |  | **49-<64** | 13.58 (16.37) 8.33 n=27 |  |  |  |
|  |  | **64-80** | 15.00 (21.15) 8.33 n=30 | -2.68 (-6.04;0.68) | 0.12 | 0.03 |
| **Sex** | 0 | **Male** | 14.58 (17.07) 8.33 n=24 |  |  |  |
|  |  | **Female** | 16.98 (19.68) 8.33 n=53 | 2.40 (-6.87;11.67) | 0.61 | 0.00 |
| **Marital status** | 0 | **Married / Cohabiting (living with a partner)** | 16.67 (19.76) 8.33 n=59 |  |  |  |
|  |  | **Living alone (single household)** | 14.81 (15.80) 8.33 n=18 | -1.85 (-12.01;8.30) | 0.72 | 0.00 |
| **Employment status** | 0 | **Employed / Student** | 16.33 (17.49) 8.33 n=50 |  |  |  |
|  |  | **Retired / Disability pension / Unemployed / No occupation** | 16.05 (21.42) 8.33 n=27 | -0.28 (-9.30;8.73) | 0.95 | 0.00 |
| **Education level** | 1 | **Compulsory school** | 25.00 (18.16) 16.67 n=9 | Reference | 0.12*** |  |
|  |  | **Upper secondary / Vocational school** | 11.81 (17.64) 8.33 n=36 | -13.19 (-26.75;0.36) | 0.056 |  |
|  |  | **University / Higher education (College)** | 17.74 (18.97) 16.67 n=31 | -7.26 (-21.03;6.52) | 0.30 | 0.06 |
| All tests are performed with univariable linear regression. P-values, Beta and R-square are based on original values and not on stratified groups ***) p-value for the entire effect/factor/variable. | | | | | | |

**Table 4.1.2 Univariable Linear Regression in** **Change of Future uncertainty (BL-60m)**

| **Variable** | **n missing** | **Value** | **Change in Future uncertainty (BL to 60m) Mean (SD) Median n=** | **Beta (95% CI)** | **p-value** | **R-Square** |
| --- | --- | --- | --- | --- | --- | --- |
| **Age (Beta per 10 units)** | 0 | **22-<49** | 2.19 (30.03) 0.00 n=19 |  |  |  |
|  |  | **49-<64** | -6.48 (21.48) -8.33 n=27 |  |  |  |
|  |  | **64-80** | -1.11 (22.40) 0.00 n=30 | -0.41 (-4.86;4.03) | 0.85 | 0.00 |
| **Sex** | 0 | **Male** | -5.43 (26.78) -8.33 n=23 |  |  |  |
|  |  | **Female** | -0.79 (23.01) 0.00 n=53 | 4.65 (-7.39;16.69) | 0.44 | 0.01 |
| **Marital status** | 0 | **Married / Cohabiting (living with a partner)** | -2.30 (23.92) 0.00 n=58 |  |  |  |
|  |  | **Living alone (single household)** | -1.85 (25.49) 0.00 n=18 | 0.45 (-12.61;13.51) | 0.95 | 0.00 |
| **Employment status** | 0 | **Employed / Student** | -3.57 (23.07) -8.33 n=49 |  |  |  |
|  |  | **Retired / Disability pension / Unemployed / No occupation** | 0.31 (26.20) 0.00 n=27 | 3.88 (-7.69;15.45) | 0.51 | 0.01 |
| **Education level** | 1 | **Compulsory school** | 11.11 (15.59) 16.67 n=9 | Reference | 0.029*** |  |
|  |  | **Upper secondary / Vocational school** | -9.76 (24.55) -8.33 n=35 | -20.87 (-37.95;-3.80) | 0.017 |  |
|  |  | **University / Higher education (College)** | 1.08 (22.64) 0.00 n=31 | -10.04 (-27.33;7.26) | 0.25 | 0.09 |
| All tests are performed with univariable linear regression. P-values, Beta and R-square are based on original values and not on stratified groups ***) p-value for the entire effect/factor/variable. | | | | | | |

**Table 4.2.1 Univariable Linear Regression of Visual disorder**

| **Variable** | **n missing** | **Value** | **Visual disorder 60m_n Mean (SD) Median n=** | **Beta (95% CI)** | **p-value** | **R-Square** |
| --- | --- | --- | --- | --- | --- | --- |
| **Age (Beta per 10 units)** | 0 | **22-<49** | 13.89 (20.98) 5.56 n=20 |  |  |  |
|  |  | **49-<64** | 13.17 (19.50) 0.00 n=27 |  |  |  |
|  |  | **64-80** | 10.74 (15.84) 0.00 n=30 | -2.53 (-5.81;0.75) | 0.13 | 0.03 |
| **Sex** | 0 | **Male** | 8.33 (14.38) 0.00 n=24 |  |  |  |
|  |  | **Female** | 14.26 (19.78) 11.11 n=53 | 5.92 (-3.04;14.89) | 0.19 | 0.02 |
| **Marital status** | 0 | **Married / Cohabiting (living with a partner)** | 12.43 (19.25) 0.00 n=59 |  |  |  |
|  |  | **Living alone (single household)** | 12.35 (15.66) 11.11 n=18 | -0.08 (-10.01;9.84) | 0.99 | 0.00 |
| **Employment status** | 0 | **Employed / Student** | 14.89 (19.52) 11.11 n=50 |  |  |  |
|  |  | **Retired / Disability pension / Unemployed / No occupation** | 7.82 (15.35) 0.00 n=27 | -7.07 (-15.72;1.58) | 0.11 | 0.03 |
| **Education level** | 1 | **Compulsory school** | 16.05 (13.73) 11.11 n=9 | Reference | 0.21*** |  |
|  |  | **Upper secondary / Vocational school** | 8.33 (11.99) 0.00 n=36 | -7.72 (-21.33;5.89) | 0.26 |  |
|  |  | **University / Higher education (College)** | 15.77 (24.47) 0.00 n=31 | -0.28 (-14.11;13.55) | 0.97 | 0.04 |
| All tests are performed with univariable linear regression. P-values, Beta and R-square are based on original values and not on stratified groups ***) p-value for the entire effect/factor/variable. | | | | | | |

**Table 4.2.2 Univariable Linear Regression in** **Change of Visual disorder (BL-60m)**

| **Variable** | **n missing** | **Value** | **Change in Visual disorder (BL to 60m) Mean (SD) Median n=** | **Beta (95% CI)** | **p-value** | **R-Square** |
| --- | --- | --- | --- | --- | --- | --- |
| **Age (Beta per 10 units)** | 0 | **22-<49** | 2.34 (23.59) 0.00 n=19 |  |  |  |
|  |  | **49-<64** | -8.64 (25.29) 0.00 n=27 |  |  |  |
|  |  | **64-80** | 1.48 (15.37) 0.00 n=30 | 0.74 (-3.25;4.74) | 0.71 | 0.00 |
| **Sex** | 0 | **Male** | -1.93 (18.85) 0.00 n=23 |  |  |  |
|  |  | **Female** | -1.89 (23.03) 0.00 n=53 | 0.05 (-10.84;10.93) | 0.99 | 0.00 |
| **Marital status** | 0 | **Married / Cohabiting (living with a partner)** | 0.96 (22.83) 0.00 n=58 |  |  |  |
|  |  | **Living alone (single household)** | -11.11 (14.76) -11.11 n=18 | -12.07 (-23.49;-0.65) | 0.039 | 0.06 |
| **Employment status** | 0 | **Employed / Student** | -4.54 (24.10) 0.00 n=49 |  |  |  |
|  |  | **Retired / Disability pension / Unemployed / No occupation** | 2.88 (15.89) 0.00 n=27 | 7.42 (-2.89;17.72) | 0.16 | 0.03 |
| **Education level** | 1 | **Compulsory school** | 6.17 (15.82) 0.00 n=9 | Reference | 0.34*** |  |
|  |  | **Upper secondary / Vocational school** | -5.40 (15.57) 0.00 n=35 | -11.57 (-27.71;4.57) | 0.16 |  |
|  |  | **University / Higher education (College)** | -1.08 (28.01) 0.00 n=31 | -7.25 (-23.60;9.10) | 0.38 | 0.03 |
| All tests are performed with univariable linear regression. P-values, Beta and R-square are based on original values and not on stratified groups ***) p-value for the entire effect/factor/variable. | | | | | | |

**Table 4.3.1 Univariable Linear Regression of** **Motor dysfunction**

| **Variable** | **n missing** | **Value** | **Motor dysfunction 60m_n Mean (SD) Median n=** | **Beta (95% CI)** | **p-value** | **R-Square** |
| --- | --- | --- | --- | --- | --- | --- |
| **Age (Beta per 10 units)** | 0 | **22-<49** | 12.22 (13.44) 11.11 n=20 |  |  |  |
|  |  | **49-<64** | 10.29 (11.91) 11.11 n=27 |  |  |  |
|  |  | **64-80** | 15.19 (16.11) 11.11 n=30 | 0.75 (-1.79;3.29) | 0.56 | 0.00 |
| **Sex** | 0 | **Male** | 12.96 (13.77) 11.11 n=24 |  |  |  |
|  |  | **Female** | 12.58 (14.30) 11.11 n=53 | -0.38 (-7.31;6.54) | 0.91 | 0.00 |
| **Marital status** | 0 | **Married / Cohabiting (living with a partner)** | 12.05 (14.49) 11.11 n=59 |  |  |  |
|  |  | **Living alone (single household)** | 14.81 (12.64) 11.11 n=18 | 2.76 (-4.79;10.32) | 0.47 | 0.01 |
| **Employment status** | 0 | **Employed / Student** | 12.89 (13.54) 11.11 n=50 |  |  |  |
|  |  | **Retired / Disability pension / Unemployed / No occupation** | 12.35 (15.20) 11.11 n=27 | -0.54 (-7.27;6.18) | 0.87 | 0.00 |
| **Education level** | 1 | **Compulsory school** | 20.99 (17.07) 22.22 n=9 | Reference | 0.083*** |  |
|  |  | **Upper secondary / Vocational school** | 13.27 (13.23) 11.11 n=36 | -7.72 (-17.97;2.54) | 0.14 |  |
|  |  | **University / Higher education (College)** | 9.32 (13.49) 0.00 n=31 | -11.67 (-22.09;-1.25) | 0.029 | 0.07 |
| All tests are performed with univariable linear regression. P-values, Beta and R-square are based on original values and not on stratified groups ***) p-value for the entire effect/factor/variable. | | | | | | |

**Table 4.3.2 Univariable Linear Regression in Change of Motor dysfunction (BL-60m)**

| **Variable** | **n missing** | **Value** | **Change in Motor dysfunction (BL to 60m) Mean (SD) Median n=** | **Beta (95% CI)** | **p-value** | **R-Square** |
| --- | --- | --- | --- | --- | --- | --- |
| **Age (Beta per 10 units)** | 0 | **22-<49** | 6.43 (10.68) 0.00 n=19 |  |  |  |
|  |  | **49-<64** | 2.47 (13.19) 0.00 n=27 |  |  |  |
|  |  | **64-80** | 9.81 (15.56) 5.56 n=30 | 1.17 (-1.37;3.70) | 0.36 | 0.01 |
| **Sex** | 0 | **Male** | 6.76 (14.11) 0.00 n=23 |  |  |  |
|  |  | **Female** | 6.18 (13.85) 0.00 n=53 | -0.58 (-7.51;6.35) | 0.87 | 0.00 |
| **Marital status** | 0 | **Married / Cohabiting (living with a partner)** | 6.23 (14.12) 0.00 n=58 |  |  |  |
|  |  | **Living alone (single household)** | 6.79 (13.28) 5.56 n=18 | 0.56 (-6.92;8.05) | 0.88 | 0.00 |
| **Employment status** | 0 | **Employed / Student** | 5.90 (13.63) 0.00 n=49 |  |  |  |
|  |  | **Retired / Disability pension / Unemployed / No occupation** | 7.20 (14.44) 0.00 n=27 | 1.31 (-5.34;7.95) | 0.70 | 0.00 |
| **Education level** | 1 | **Compulsory school** | 12.35 (17.07) 11.11 n=9 | Reference | 0.16*** |  |
|  |  | **Upper secondary / Vocational school** | 7.30 (13.99) 0.00 n=35 | -5.04 (-15.22;5.13) | 0.33 |  |
|  |  | **University / Higher education (College)** | 3.05 (12.16) 0.00 n=31 | -9.30 (-19.60;1.01) | 0.076 | 0.05 |
| All tests are performed with univariable linear regression. P-values, Beta and R-square are based on original values and not on stratified groups ***) p-value for the entire effect/factor/variable. | | | | | | |

**Table 4.4.1 Univariable Linear Regression of Communication deficit**

| **Variable** | **n missing** | **Value** | **Communication deficit 60m_n Mean (SD) Median n=** | **Beta (95% CI)** | **p-value** | **R-Square** |
| --- | --- | --- | --- | --- | --- | --- |
| **Age (Beta per 10 units)** | 0 | **22-<49** | 18.89 (22.83) 16.67 n=20 |  |  |  |
|  |  | **49-<64** | 7.82 (10.13) 0.00 n=27 |  |  |  |
|  |  | **64-80** | 12.22 (14.98) 5.56 n=30 | -2.39 (-5.32;0.53) | 0.11 | 0.03 |
| **Sex** | 0 | **Male** | 13.89 (14.38) 11.11 n=24 |  |  |  |
|  |  | **Female** | 11.74 (17.35) 0.00 n=53 | -2.15 (-10.23;5.94) | 0.60 | 0.00 |
| **Marital status** | 0 | **Married / Cohabiting (living with a partner)** | 12.05 (16.67) 0.00 n=59 |  |  |  |
|  |  | **Living alone (single household)** | 13.58 (15.97) 11.11 n=18 | 1.53 (-7.33;10.39) | 0.73 | 0.00 |
| **Employment status** | 0 | **Employed / Student** | 12.67 (17.24) 5.56 n=50 |  |  |  |
|  |  | **Retired / Disability pension / Unemployed / No occupation** | 11.93 (15.07) 0.00 n=27 | -0.73 (-8.59;7.13) | 0.85 | 0.00 |
| **Education level** | 1 | **Compulsory school** | 18.52 (18.43) 22.22 n=9 | Reference | 0.49*** |  |
|  |  | **Upper secondary / Vocational school** | 11.42 (12.87) 11.11 n=36 | -7.10 (-19.39;5.19) | 0.25 |  |
|  |  | **University / Higher education (College)** | 11.47 (19.56) 0.00 n=31 | -7.05 (-19.54;5.44) | 0.26 | 0.02 |
| All tests are performed with univariable linear regression. P-values, Beta and R-square are based on original values and not on stratified groups ***) p-value for the entire effect/factor/variable. | | | | | | |

**Table 4.4.2 Univariable Linear Regression in** **Change of Communication deficit (BL-60m)**

| **Variable** | **n missing** | **Value** | **Change in Communication deficit (BL to 60m) Mean (SD) Median n=** | **Beta (95% CI)** | **p-value** | **R-Square** |
| --- | --- | --- | --- | --- | --- | --- |
| **Age (Beta per 10 units)** | 0 | **22-<49** | 11.70 (16.34) 11.11 n=19 |  |  |  |
|  |  | **49-<64** | 1.23 (9.91) 0.00 n=27 |  |  |  |
|  |  | **64-80** | 2.59 (15.63) 0.00 n=30 | -2.36 (-4.98;0.26) | 0.076 | 0.04 |
| **Sex** | 0 | **Male** | 10.14 (17.05) 11.11 n=23 |  |  |  |
|  |  | **Female** | 1.89 (12.66) 0.00 n=53 | -8.26 (-15.28;-1.24) | 0.022 | 0.07 |
| **Marital status** | 0 | **Married / Cohabiting (living with a partner)** | 4.79 (14.29) 0.00 n=58 |  |  |  |
|  |  | **Living alone (single household)** | 3.09 (15.62) 0.00 n=18 | -1.70 (-9.55;6.15) | 0.67 | 0.00 |
| **Employment status** | 0 | **Employed / Student** | 4.31 (14.31) 0.00 n=49 |  |  |  |
|  |  | **Retired / Disability pension / Unemployed / No occupation** | 4.53 (15.18) 0.00 n=27 | 0.22 (-6.76;7.20) | 0.95 | 0.00 |
| **Education level** | 1 | **Compulsory school** | 6.17 (20.12) 11.11 n=9 | Reference | 0.67*** |  |
|  |  | **Upper secondary / Vocational school** | 2.54 (13.23) 0.00 n=35 | -3.63 (-14.50;7.24) | 0.51 |  |
|  |  | **University / Higher education (College)** | 5.38 (14.31) 0.00 n=31 | -0.80 (-11.81;10.22) | 0.89 | 0.01 |
| All tests are performed with univariable linear regression. P-values, Beta and R-square are based on original values and not on stratified groups ***) p-value for the entire effect/factor/variable. | | | | | | |

**Table 4.5.1 Univariable Linear Regression of** **Headaches**

| **Variable** | **n missing** | **Value** | **Headaches 60m_n Mean (SD) Median n=** | **Beta (95% CI)** | **p-value** | **R-Square** |
| --- | --- | --- | --- | --- | --- | --- |
| **Age (Beta per 10 units)** | 0 | **22-<49** | 31.67 (27.52) 33.33 n=20 |  |  |  |
|  |  | **49-<64** | 22.22 (32.03) 0.00 n=27 |  |  |  |
|  |  | **64-80** | 11.11 (18.22) 0.00 n=30 | -7.15 (-11.78;-2.52) | 0.0029 | 0.11 |
| **Sex** | 0 | **Male** | 18.06 (24.04) 0.00 n=24 |  |  |  |
|  |  | **Female** | 21.38 (28.57) 0.00 n=53 | 3.33 (-10.03;16.69) | 0.62 | 0.00 |
| **Marital status** | 0 | **Married / Cohabiting (living with a partner)** | 20.34 (26.99) 0.00 n=59 |  |  |  |
|  |  | **Living alone (single household)** | 20.37 (28.33) 0.00 n=18 | 0.03 (-14.61;14.68) | 1.00 | 0.00 |
| **Employment status** | 0 | **Employed / Student** | 24.67 (29.97) 16.67 n=50 |  |  |  |
|  |  | **Retired / Disability pension / Unemployed / No occupation** | 12.35 (18.83) 0.00 n=27 | -12.32 (-25.00;0.35) | 0.057 | 0.05 |
| **Education level** | 1 | **Compulsory school** | 11.11 (16.67) 0.00 n=9 | Reference | 0.60*** |  |
|  |  | **Upper secondary / Vocational school** | 20.37 (26.76) 0.00 n=36 | 9.26 (-9.94;28.46) | 0.34 |  |
|  |  | **University / Higher education (College)** | 20.43 (26.77) 0.00 n=31 | 9.32 (-10.19;28.83) | 0.34 | 0.01 |
| All tests are performed with univariable linear regression. P-values, Beta and R-square are based on original values and not on stratified groups ***) p-value for the entire effect/factor/variable. | | | | | | |

**Table 4.5.2 Univariable Linear Regression in Change of Headaches (BL-60m)**

| **Variable** | **n missing** | **Value** | **Change in Headaches (BL to 60m) Mean (SD) Median n=** | **Beta (95% CI)** | **p-value** | **R-Square** |
| --- | --- | --- | --- | --- | --- | --- |
| **Age (Beta per 10 units)** | 0 | **22-<49** | 3.51 (18.90) 0.00 n=19 |  |  |  |
|  |  | **49-<64** | -4.94 (27.27) 0.00 n=27 |  |  |  |
|  |  | **64-80** | -11.11 (26.74) 0.00 n=30 | -3.41 (-8.05;1.23) | 0.15 | 0.03 |
| **Sex** | 0 | **Male** | -1.45 (23.52) 0.00 n=23 |  |  |  |
|  |  | **Female** | -6.92 (26.44) 0.00 n=53 | -5.47 (-18.21;7.27) | 0.40 | 0.01 |
| **Marital status** | 0 | **Married / Cohabiting (living with a partner)** | -5.17 (26.34) 0.00 n=58 |  |  |  |
|  |  | **Living alone (single household)** | -5.56 (23.57) 0.00 n=18 | -0.38 (-14.22;13.45) | 0.96 | 0.00 |
| **Employment status** | 0 | **Employed / Student** | -4.08 (27.75) 0.00 n=49 |  |  |  |
|  |  | **Retired / Disability pension / Unemployed / No occupation** | -7.41 (21.35) 0.00 n=27 | -3.33 (-15.59;8.94) | 0.59 | 0.00 |
| **Education level** | 1 | **Compulsory school** | -7.41 (14.70) 0.00 n=9 | Reference | 0.89*** |  |
|  |  | **Upper secondary / Vocational school** | -4.76 (31.46) 0.00 n=35 | 2.65 (-15.69;20.98) | 0.77 |  |
|  |  | **University / Higher education (College)** | -7.53 (16.58) 0.00 n=31 | -0.12 (-18.70;18.46) | 0.99 | 0.00 |
| All tests are performed with univariable linear regression. P-values, Beta and R-square are based on original values and not on stratified groups ***) p-value for the entire effect/factor/variable. | | | | | | |

**Table 4.6.1 Univariable Linear Regression of** **Seizures**

| **Variable** | **n missing** | **Value** | **Seizures 60m_n Mean (SD) Median n=** | **Beta (95% CI)** | **p-value** | **R-Square** |
| --- | --- | --- | --- | --- | --- | --- |
| **Age (Beta per 10 units)** | 0 | **22-<49** | 1.67 (7.45) 0.00 n=20 |  |  |  |
|  |  | **49-<64** | 1.23 (6.42) 0.00 n=27 |  |  |  |
|  |  | **64-80** | 0.00 (0.00) 0.00 n=30 | -0.85 (-1.79;0.10) | 0.079 | 0.04 |
| **Sex** | 0 | **Male** | 1.39 (6.80) 0.00 n=24 |  |  |  |
|  |  | **Female** | 0.63 (4.58) 0.00 n=53 | -0.76 (-3.39;1.87) | 0.57 | 0.00 |
| **Marital status** | 0 | **Married / Cohabiting (living with a partner)** | 0.00 (0.00) 0.00 n=59 |  |  |  |
|  |  | **Living alone (single household)** | 3.70 (10.78) 0.00 n=18 | 3.70 (0.95;6.46) | 0.0090 | 0.09 |
| **Employment status** | 0 | **Employed / Student** | 1.33 (6.60) 0.00 n=50 |  |  |  |
|  |  | **Retired / Disability pension / Unemployed / No occupation** | 0.00 (0.00) 0.00 n=27 | -1.33 (-3.87;1.20) | 0.30 | 0.01 |
| **Education level** | 1 | **Compulsory school** | 0.00 (0.00) 0.00 n=9 | Reference | 0.87*** |  |
|  |  | **Upper secondary / Vocational school** | 0.93 (5.56) 0.00 n=36 | 0.93 (-3.11;4.96) | 0.65 |  |
|  |  | **University / Higher education (College)** | 1.08 (5.99) 0.00 n=31 | 1.08 (-3.03;5.18) | 0.60 | 0.00 |
| All tests are performed with univariable linear regression. P-values, Beta and R-square are based on original values and not on stratified groups ***) p-value for the entire effect/factor/variable. | | | | | | |

**Table 4.6.2 Univariable Linear Regression in** **Change of Seizures (BL-60m)**

| **Variable** | **n missing** | **Value** | **Change in Seizures (BL to 60m) Mean (SD) Median n=** | **Beta (95% CI)** | **p-value** | **R-Square** |
| --- | --- | --- | --- | --- | --- | --- |
| **Age (Beta per 10 units)** | 0 | **22-<49** | -3.51 (15.29) 0.00 n=19 |  |  |  |
|  |  | **49-<64** | 1.23 (6.42) 0.00 n=27 |  |  |  |
|  |  | **64-80** | -1.11 (6.09) 0.00 n=30 | 0.64 (-1.09;2.36) | 0.46 | 0.01 |
| **Sex** | 0 | **Male** | -4.35 (15.26) 0.00 n=23 |  |  |  |
|  |  | **Female** | 0.63 (4.58) 0.00 n=53 | 4.98 (0.42;9.53) | 0.033 | 0.06 |
| **Marital status** | 0 | **Married / Cohabiting (living with a partner)** | -1.72 (9.72) 0.00 n=58 |  |  |  |
|  |  | **Living alone (single household)** | 1.85 (7.86) 0.00 n=18 | 3.58 (-1.44;8.59) | 0.16 | 0.03 |
| **Employment status** | 0 | **Employed / Student** | -0.68 (10.74) 0.00 n=49 |  |  |  |
|  |  | **Retired / Disability pension / Unemployed / No occupation** | -1.23 (6.42) 0.00 n=27 | -0.55 (-5.07;3.96) | 0.81 | 0.00 |
| **Education level** | 1 | **Compulsory school** | -3.70 (11.11) 0.00 n=9 | Reference | 0.58*** |  |
|  |  | **Upper secondary / Vocational school** | 0.00 (0.00) 0.00 n=35 | 3.70 (-3.38;10.79) | 0.30 |  |
|  |  | **University / Higher education (College)** | -1.08 (13.56) 0.00 n=31 | 2.63 (-4.55;9.80) | 0.47 | 0.02 |
| All tests are performed with univariable linear regression. P-values, Beta and R-square are based on original values and not on stratified groups ***) p-value for the entire effect/factor/variable. | | | | | | |

**Table 4.7.1 Univariable Linear Regression of** **Drowsiness**

| **Variable** | **n missing** | **Value** | **Drowsiness 60m_n Mean (SD) Median n=** | **Beta (95% CI)** | **p-value** | **R-Square** |
| --- | --- | --- | --- | --- | --- | --- |
| **Age (Beta per 10 units)** | 0 | **22-<49** | 40.00 (31.72) 33.33 n=20 |  |  |  |
|  |  | **49-<64** | 38.27 (28.80) 33.33 n=27 |  |  |  |
|  |  | **64-80** | 35.56 (27.59) 33.33 n=30 | -1.86 (-7.06;3.34) | 0.48 | 0.01 |
| **Sex** | 0 | **Male** | 40.28 (29.45) 33.33 n=24 |  |  |  |
|  |  | **Female** | 36.48 (28.69) 33.33 n=53 | -3.80 (-17.98;10.38) | 0.60 | 0.00 |
| **Marital status** | 0 | **Married / Cohabiting (living with a partner)** | 36.16 (29.87) 33.33 n=59 |  |  |  |
|  |  | **Living alone (single household)** | 42.59 (25.06) 33.33 n=18 | 6.43 (-9.04;21.91) | 0.41 | 0.01 |
| **Employment status** | 0 | **Employed / Student** | 38.00 (29.36) 33.33 n=50 |  |  |  |
|  |  | **Retired / Disability pension / Unemployed / No occupation** | 37.04 (28.24) 33.33 n=27 | -0.96 (-14.75;12.82) | 0.89 | 0.00 |
| **Education level** | 1 | **Compulsory school** | 59.26 (27.78) 66.67 n=9 | Reference | 0.049*** |  |
|  |  | **Upper secondary / Vocational school** | 34.26 (25.80) 33.33 n=36 | -25.00 (-45.79;-4.21) | 0.019 |  |
|  |  | **University / Higher education (College)** | 34.41 (30.41) 33.33 n=31 | -24.85 (-45.98;-3.72) | 0.022 | 0.08 |
| All tests are performed with univariable linear regression. P-values, Beta and R-square are based on original values and not on stratified groups ***) p-value for the entire effect/factor/variable. | | | | | | |

**Table 4.7.2 Univariable Linear Regression in** **Change of Drowsiness (BL-60m)**

| **Variable** | **n missing** | **Value** | **Change in Drowsiness (BL to 60m) Mean (SD) Median n=** | **Beta (95% CI)** | **p-value** | **R-Square** |
| --- | --- | --- | --- | --- | --- | --- |
| **Age (Beta per 10 units)** | 0 | **22-<49** | 7.02 (30.59) 0.00 n=19 |  |  |  |
|  |  | **49-<64** | 7.41 (29.72) 0.00 n=27 |  |  |  |
|  |  | **64-80** | 13.33 (28.50) 0.00 n=30 | 1.58 (-3.79;6.95) | 0.56 | 0.00 |
| **Sex** | 0 | **Male** | 10.14 (32.47) 0.00 n=23 |  |  |  |
|  |  | **Female** | 9.43 (28.02) 0.00 n=53 | -0.71 (-15.35;13.92) | 0.92 | 0.00 |
| **Marital status** | 0 | **Married / Cohabiting (living with a partner)** | 9.77 (31.22) 0.00 n=58 |  |  |  |
|  |  | **Living alone (single household)** | 9.26 (22.30) 0.00 n=18 | -0.51 (-16.32;15.30) | 0.95 | 0.00 |
| **Employment status** | 0 | **Employed / Student** | 6.80 (29.64) 0.00 n=49 |  |  |  |
|  |  | **Retired / Disability pension / Unemployed / No occupation** | 14.81 (28.24) 0.00 n=27 | 8.01 (-5.91;21.94) | 0.26 | 0.02 |
| **Education level** | 1 | **Compulsory school** | 29.63 (26.06) 33.33 n=9 | Reference | 0.045*** |  |
|  |  | **Upper secondary / Vocational school** | 2.86 (24.75) 0.00 n=35 | -26.77 (-47.96;-5.58) | 0.014 |  |
|  |  | **University / Higher education (College)** | 10.75 (32.65) 0.00 n=31 | -18.88 (-40.34;2.59) | 0.084 | 0.08 |
| All tests are performed with univariable linear regression. P-values, Beta and R-square are based on original values and not on stratified groups ***) p-value for the entire effect/factor/variable. | | | | | | |

**Table 4.8.1 Univariable Linear Regression of Itchy skin**

| **Variable** | **n missing** | **Value** | **Itchy skin 60m_n Mean (SD) Median n=** | **Beta (95% CI)** | **p-value** | **R-Square** |
| --- | --- | --- | --- | --- | --- | --- |
| **Age (Beta per 10 units)** | 0 | **22-<49** | 10.00 (15.67) 0.00 n=20 |  |  |  |
|  |  | **49-<64** | 12.35 (18.83) 0.00 n=27 |  |  |  |
|  |  | **64-80** | 12.22 (22.29) 0.00 n=30 | 1.37 (-2.12;4.86) | 0.44 | 0.01 |
| **Sex** | 0 | **Male** | 11.11 (25.38) 0.00 n=24 |  |  |  |
|  |  | **Female** | 11.95 (16.14) 0.00 n=53 | 0.84 (-8.69;10.37) | 0.86 | 0.00 |
| **Marital status** | 0 | **Married / Cohabiting (living with a partner)** | 10.73 (20.00) 0.00 n=59 |  |  |  |
|  |  | **Living alone (single household)** | 14.81 (17.04) 0.00 n=18 | 4.08 (-6.31;14.47) | 0.44 | 0.01 |
| **Employment status** | 0 | **Employed / Student** | 11.33 (17.31) 0.00 n=50 |  |  |  |
|  |  | **Retired / Disability pension / Unemployed / No occupation** | 12.35 (22.92) 0.00 n=27 | 1.01 (-8.24;10.26) | 0.83 | 0.00 |
| **Education level** | 1 | **Compulsory school** | 29.63 (35.14) 33.33 n=9 | Reference | 0.005*** |  |
|  |  | **Upper secondary / Vocational school** | 11.11 (15.94) 0.00 n=36 | -18.52 (-32.03;-5.01) | 0.0079 |  |
|  |  | **University / Higher education (College)** | 6.45 (13.39) 0.00 n=31 | -23.18 (-36.90;-9.45) | 0.0012 | 0.13 |
| All tests are performed with univariable linear regression. P-values, Beta and R-square are based on original values and not on stratified groups ***) p-value for the entire effect/factor/variable. | | | | | | |

**Table 4.8.2 Univariable Linear Regression in Change of Itchy skin (BL-60m)**

| **Variable** | **n missing** | **Value** | **Change in Itchy skin (BL to 60m) Mean (SD) Median n=** | **Beta (95% CI)** | **p-value** | **R-Square** |
| --- | --- | --- | --- | --- | --- | --- |
| **Age (Beta per 10 units)** | 0 | **22-<49** | 5.26 (16.72) 0.00 n=19 |  |  |  |
|  |  | **49-<64** | 8.64 (19.81) 0.00 n=27 |  |  |  |
|  |  | **64-80** | 7.78 (28.61) 0.00 n=30 | 1.78 (-2.41;5.97) | 0.40 | 0.01 |
| **Sex** | 0 | **Male** | 10.14 (27.40) 0.00 n=23 |  |  |  |
|  |  | **Female** | 6.29 (20.73) 0.00 n=53 | -3.86 (-15.26;7.55) | 0.50 | 0.01 |
| **Marital status** | 0 | **Married / Cohabiting (living with a partner)** | 6.90 (23.99) 0.00 n=58 |  |  |  |
|  |  | **Living alone (single household)** | 9.26 (19.15) 0.00 n=18 | 2.36 (-9.98;14.71) | 0.70 | 0.00 |
| **Employment status** | 0 | **Employed / Student** | 7.48 (18.34) 0.00 n=49 |  |  |  |
|  |  | **Retired / Disability pension / Unemployed / No occupation** | 7.41 (29.72) 0.00 n=27 | -0.08 (-11.05;10.90) | 0.99 | 0.00 |
| **Education level** | 1 | **Compulsory school** | 29.63 (35.14) 33.33 n=9 | Reference | 0.004*** |  |
|  |  | **Upper secondary / Vocational school** | 5.71 (20.59) 0.00 n=35 | -23.92 (-39.87;-7.96) | 0.0038 |  |
|  |  | **University / Higher education (College)** | 2.15 (17.07) 0.00 n=31 | -27.48 (-43.65;-11.31) | 0.0011 | 0.14 |
| All tests are performed with univariable linear regression. P-values, Beta and R-square are based on original values and not on stratified groups ***) p-value for the entire effect/factor/variable. | | | | | | |

**Table 4.9.1 Univariable Linear Regression of Hair loss**

| **Variable** | **n missing** | **Value** | **Hair loss 60m_n Mean (SD) Median n=** | **Beta (95% CI)** | **p-value** | **R-Square** |
| --- | --- | --- | --- | --- | --- | --- |
| **Age (Beta per 10 units)** | 0 | **22-<49** | 6.67 (17.44) 0.00 n=20 |  |  |  |
|  |  | **49-<64** | 12.35 (20.98) 0.00 n=27 |  |  |  |
|  |  | **64-80** | 15.56 (24.34) 0.00 n=30 | 3.23 (-0.61;7.07) | 0.098 | 0.04 |
| **Sex** | 0 | **Male** | 6.94 (16.97) 0.00 n=24 |  |  |  |
|  |  | **Female** | 14.47 (23.12) 0.00 n=53 | 7.52 (-2.98;18.02) | 0.16 | 0.03 |
| **Marital status** | 0 | **Married / Cohabiting (living with a partner)** | 11.30 (22.84) 0.00 n=59 |  |  |  |
|  |  | **Living alone (single household)** | 14.81 (17.04) 0.00 n=18 | 3.52 (-8.10;15.13) | 0.55 | 0.00 |
| **Employment status** | 0 | **Employed / Student** | 9.33 (19.10) 0.00 n=50 |  |  |  |
|  |  | **Retired / Disability pension / Unemployed / No occupation** | 17.28 (25.10) 0.00 n=27 | 7.95 (-2.22;18.12) | 0.12 | 0.03 |
| **Education level** | 1 | **Compulsory school** | 14.81 (17.57) 0.00 n=9 | Reference | 0.79*** |  |
|  |  | **Upper secondary / Vocational school** | 12.04 (22.75) 0.00 n=36 | -2.78 (-18.36;12.80) | 0.72 |  |
|  |  | **University / Higher education (College)** | 9.68 (19.61) 0.00 n=31 | -5.14 (-20.97;10.69) | 0.52 | 0.01 |
| All tests are performed with univariable linear regression. P-values, Beta and R-square are based on original values and not on stratified groups ***) p-value for the entire effect/factor/variable. | | | | | | |

**Table 4.9.2 Univariable Linear Regression in Change of Hair loss (BL-60m)**

| **Variable** | **n missing** | **Value** | **Change in Hair loss (BL to 60m) Mean (SD) Median n=** | **Beta (95% CI)** | **p-value** | **R-Square** |
| --- | --- | --- | --- | --- | --- | --- |
| **Age (Beta per 10 units)** | 0 | **22-<49** | 5.26 (20.07) 0.00 n=19 |  |  |  |
|  |  | **49-<64** | 9.88 (24.13) 0.00 n=27 |  |  |  |
|  |  | **64-80** | 15.56 (24.34) 0.00 n=30 | 3.48 (-0.74;7.71) | 0.10 | 0.04 |
| **Sex** | 0 | **Male** | 5.80 (19.21) 0.00 n=23 |  |  |  |
|  |  | **Female** | 13.21 (24.75) 0.00 n=53 | 7.41 (-4.15;18.97) | 0.21 | 0.02 |
| **Marital status** | 0 | **Married / Cohabiting (living with a partner)** | 10.34 (24.35) 0.00 n=58 |  |  |  |
|  |  | **Living alone (single household)** | 12.96 (20.26) 0.00 n=18 | 2.62 (-10.00;15.24) | 0.68 | 0.00 |
| **Employment status** | 0 | **Employed / Student** | 7.48 (21.80) 0.00 n=49 |  |  |  |
|  |  | **Retired / Disability pension / Unemployed / No occupation** | 17.28 (25.10) 0.00 n=27 | 9.80 (-1.19;20.79) | 0.080 | 0.04 |
| **Education level** | 1 | **Compulsory school** | 14.81 (17.57) 0.00 n=9 | Reference | 0.77*** |  |
|  |  | **Upper secondary / Vocational school** | 10.48 (25.27) 0.00 n=35 | -4.34 (-21.33;12.65) | 0.61 |  |
|  |  | **University / Higher education (College)** | 8.60 (21.03) 0.00 n=31 | -6.21 (-23.43;11.00) | 0.47 | 0.01 |
| All tests are performed with univariable linear regression. P-values, Beta and R-square are based on original values and not on stratified groups ***) p-value for the entire effect/factor/variable. | | | | | | |

**Table 4.10.1 Univariable Linear Regression of Weakness of legs**

| **Variable** | **n missing** | **Value** | **Weakness of legs 60m_n Mean (SD) Median n=** | **Beta (95% CI)** | **p-value** | **R-Square** |
| --- | --- | --- | --- | --- | --- | --- |
| **Age (Beta per 10 units)** | 0 | **22-<49** | 6.67 (17.44) 0.00 n=20 |  |  |  |
|  |  | **49-<64** | 7.41 (14.12) 0.00 n=27 |  |  |  |
|  |  | **64-80** | 11.11 (22.03) 0.00 n=30 | 1.34 (-1.96;4.63) | 0.42 | 0.01 |
| **Sex** | 0 | **Male** | 5.56 (12.69) 0.00 n=24 |  |  |  |
|  |  | **Female** | 10.06 (20.24) 0.00 n=53 | 4.51 (-4.44;13.46) | 0.32 | 0.01 |
| **Marital status** | 0 | **Married / Cohabiting (living with a partner)** | 7.91 (17.87) 0.00 n=59 |  |  |  |
|  |  | **Living alone (single household)** | 11.11 (19.80) 0.00 n=18 | 3.20 (-6.63;13.03) | 0.52 | 0.01 |
| **Employment status** | 0 | **Employed / Student** | 8.00 (17.25) 0.00 n=50 |  |  |  |
|  |  | **Retired / Disability pension / Unemployed / No occupation** | 9.88 (20.29) 0.00 n=27 | 1.88 (-6.86;10.61) | 0.67 | 0.00 |
| **Education level** | 1 | **Compulsory school** | 14.81 (17.57) 0.00 n=9 | Reference | 0.56*** |  |
|  |  | **Upper secondary / Vocational school** | 7.41 (18.01) 0.00 n=36 | -7.41 (-21.12;6.30) | 0.29 |  |
|  |  | **University / Higher education (College)** | 8.60 (19.18) 0.00 n=31 | -6.21 (-20.14;7.72) | 0.38 | 0.02 |
| All tests are performed with univariable linear regression. P-values, Beta and R-square are based on original values and not on stratified groups ***) p-value for the entire effect/factor/variable. | | | | | | |

**Table 4.10.2 Univariable Linear Regression in Change of Weakness of legs (BL-60m)**

| **Variable** | **n missing** | **Value** | **Change in Weakness of legs (BL to 60m) Mean (SD) Median n=** | **Beta (95% CI)** | **p-value** | **R-Square** |
| --- | --- | --- | --- | --- | --- | --- |
| **Age (Beta per 10 units)** | 0 | **22-<49** | 3.51 (18.90) 0.00 n=19 |  |  |  |
|  |  | **49-<64** | 2.47 (20.52) 0.00 n=27 |  |  |  |
|  |  | **64-80** | 8.89 (21.32) 0.00 n=30 | 1.08 (-2.67;4.83) | 0.57 | 0.00 |
| **Sex** | 0 | **Male** | 0.00 (17.41) 0.00 n=23 |  |  |  |
|  |  | **Female** | 7.55 (21.33) 0.00 n=53 | 7.55 (-2.52;17.62) | 0.14 | 0.03 |
| **Marital status** | 0 | **Married / Cohabiting (living with a partner)** | 4.60 (21.13) 0.00 n=58 |  |  |  |
|  |  | **Living alone (single household)** | 7.41 (18.28) 0.00 n=18 | 2.81 (-8.21;13.83) | 0.61 | 0.00 |
| **Employment status** | 0 | **Employed / Student** | 4.08 (21.12) 0.00 n=49 |  |  |  |
|  |  | **Retired / Disability pension / Unemployed / No occupation** | 7.41 (19.25) 0.00 n=27 | 3.33 (-6.45;13.11) | 0.50 | 0.01 |
| **Education level** | 1 | **Compulsory school** | 3.70 (11.11) 0.00 n=9 | Reference | 0.92*** |  |
|  |  | **Upper secondary / Vocational school** | 4.76 (23.07) 0.00 n=35 | 1.06 (-14.43;16.55) | 0.89 |  |
|  |  | **University / Higher education (College)** | 6.45 (20.04) 0.00 n=31 | 2.75 (-12.95;18.44) | 0.73 | 0.00 |
| All tests are performed with univariable linear regression. P-values, Beta and R-square are based on original values and not on stratified groups ***) p-value for the entire effect/factor/variable. | | | | | | |

**Table 4.11.1 Univariable Linear Regression of Bladder control**

| **Variable** | **n missing** | **Value** | **Bladder control 60m_n Mean (SD) Median n=** | **Beta (95% CI)** | **p-value** | **R-Square** |
| --- | --- | --- | --- | --- | --- | --- |
| **Age (Beta per 10 units)** | 0 | **22-<49** | 5.00 (12.21) 0.00 n=20 |  |  |  |
|  |  | **49-<64** | 9.88 (22.29) 0.00 n=27 |  |  |  |
|  |  | **64-80** | 20.00 (28.50) 0.00 n=30 | 5.78 (1.71;9.84) | 0.0059 | 0.10 |
| **Sex** | 0 | **Male** | 13.89 (27.66) 0.00 n=24 |  |  |  |
|  |  | **Female** | 11.95 (21.77) 0.00 n=53 | -1.94 (-13.57;9.69) | 0.74 | 0.00 |
| **Marital status** | 0 | **Married / Cohabiting (living with a partner)** | 12.43 (24.68) 0.00 n=59 |  |  |  |
|  |  | **Living alone (single household)** | 12.96 (20.26) 0.00 n=18 | 0.53 (-12.21;13.27) | 0.93 | 0.00 |
| **Employment status** | 0 | **Employed / Student** | 8.67 (18.82) 0.00 n=50 |  |  |  |
|  |  | **Retired / Disability pension / Unemployed / No occupation** | 19.75 (29.61) 0.00 n=27 | 11.09 (0.08;22.09) | 0.048 | 0.05 |
| **Education level** | 1 | **Compulsory school** | 25.93 (32.39) 0.00 n=9 | Reference | 0.071*** |  |
|  |  | **Upper secondary / Vocational school** | 14.81 (26.96) 0.00 n=36 | -11.11 (-28.32;6.10) | 0.20 |  |
|  |  | **University / Higher education (College)** | 6.45 (13.39) 0.00 n=31 | -19.47 (-36.96;-1.99) | 0.030 | 0.07 |
| All tests are performed with univariable linear regression. P-values, Beta and R-square are based on original values and not on stratified groups ***) p-value for the entire effect/factor/variable. | | | | | | |

**Table 4.11.2 Univariable Linear Regression in Change of Bladder control (BL-60m)**

| **Variable** | **n missing** | **Value** | **Change in Bladder control (BL to 60m) Mean (SD) Median n=** | **Beta (95% CI)** | **p-value** | **R-Square** |
| --- | --- | --- | --- | --- | --- | --- |
| **Age (Beta per 10 units)** | 0 | **22-<49** | 1.75 (13.49) 0.00 n=19 |  |  |  |
|  |  | **49-<64** | 4.94 (23.94) 0.00 n=27 |  |  |  |
|  |  | **64-80** | 13.33 (28.50) 0.00 n=30 | 4.48 (0.16;8.80) | 0.042 | 0.05 |
| **Sex** | 0 | **Male** | 7.25 (30.08) 0.00 n=23 |  |  |  |
|  |  | **Female** | 7.55 (21.33) 0.00 n=53 | 0.30 (-11.77;12.37) | 0.96 | 0.00 |
| **Marital status** | 0 | **Married / Cohabiting (living with a partner)** | 8.05 (21.91) 0.00 n=58 |  |  |  |
|  |  | **Living alone (single household)** | 5.56 (30.78) 0.00 n=18 | -2.49 (-15.52;10.54) | 0.70 | 0.00 |
| **Employment status** | 0 | **Employed / Student** | 4.76 (20.41) 0.00 n=49 |  |  |  |
|  |  | **Retired / Disability pension / Unemployed / No occupation** | 12.35 (29.45) 0.00 n=27 | 7.58 (-3.87;19.04) | 0.19 | 0.02 |
| **Education level** | 1 | **Compulsory school** | 22.22 (37.27) 0.00 n=9 | Reference | 0.085*** |  |
|  |  | **Upper secondary / Vocational school** | 8.57 (27.23) 0.00 n=35 | -13.65 (-31.35;4.05) | 0.13 |  |
|  |  | **University / Higher education (College)** | 2.15 (11.97) 0.00 n=31 | -20.07 (-38.00;-2.14) | 0.029 | 0.07 |
| All tests are performed with univariable linear regression. P-values, Beta and R-square are based on original values and not on stratified groups ***) p-value for the entire effect/factor/variable. | | | | | | |

**Figure 1 Kaplan-Meier**
